# Transcriptome profiling and weighted gene co-expression network analysis of early floral development in *Aquilegia coerulea*

**DOI:** 10.1038/s41598-020-76750-7

**Published:** 2020-11-12

**Authors:** Ya Min, Elena M. Kramer

**Affiliations:** Department of Organismic and Evolutionary Biology, Harvard University, 16 Divinity Ave., Cambridge, MA USA

**Keywords:** Morphogenesis, Stem cells, Plant development, Plant molecular biology, Plant reproduction

## Abstract

The earliest phases of floral development include a number of crucial processes that lay the foundation for the subsequent morphogenesis of floral organs and success in reproduction. Currently, key transcriptional changes during this developmental window have been characterized in the model species *Arabidopsis thaliana*, but little is known about how transcriptional dynamics change over the course of these developmental processes in other plant systems. Here, we have conducted the first in-depth transcriptome profiling of early floral development in *Aquilegia* at four finely dissected developmental stages, with eight biological replicates per stage. Using differential gene expression analysis and weighted gene co-expression network analysis, we identified both crucial genes whose expression changes mark the transitions between developmental stages and hub genes in co-expression modules. Our results support the potential functional conservation of key genes in early floral development that have been identified in other systems, but also reveal a number of previously unknown or overlooked loci that are worthy of further investigation. In addition, our results highlight not only the dynamics of transcriptional regulation during early floral development, but also the potential involvement of the complex, essential networks of small RNA and post-translational regulation to these developmental stages.

## Introduction

The earliest phase of floral meristem (FM) development requires exquisite coordination among many different developmental processes. These include the proper initiation and patterning of the floral organs, maintenance of the size of the FM during organ initiation, the eventual termination of the FM activity to ensure the correct number of whorls, and the overlay of the floral organ identity programs onto the primordia so that the boundaries of gene expression domains synchronize precisely with the physical boundaries between the primordia. Coordination of these processes is achieved through crosstalk between numerous regulatory frameworks at multiple levels, ranging from transcriptional regulation, to RNA stability, to epigenetic modification, and protein stability.

During the past 30 years, key regulatory mechanisms involved in early floral development have been characterized thanks to in-depth studies of model species such as *Arabidopsis thaliana*. These include the ABCE model of organ identity^1,2^, the *WUSCHEL* (*WUS*)-*CLAVATA* (*CLV*) feedback loop for meristem maintenance^3^, the *AGAMOUS* (*AG*)-*KNUCKLES* (*KNU*)-*WUS* pathway for FM termination^4,5^, the auxin signaling pathway for primordia initiation^6^, as well as genes specifying the boundaries between organs or gene expression domains^7^. However, novel genes and pathways contributing to these processes are constantly being discovered, and it is not yet clear how cross-regulation between different pathways and between different levels of the regulatory framework is controlled. Furthermore, although the orthologs of many genes have been studied in multiple plant model systems, relatively little is known about the degree of conservation of these programs across major lineages of flowering plants. To obtain a comprehensive view of the molecular basis of the early floral development, the conventional forward and reverse genetics approaches need to be coupled with global analysis at the transcriptional and genomic levels.

**Figure 1:**
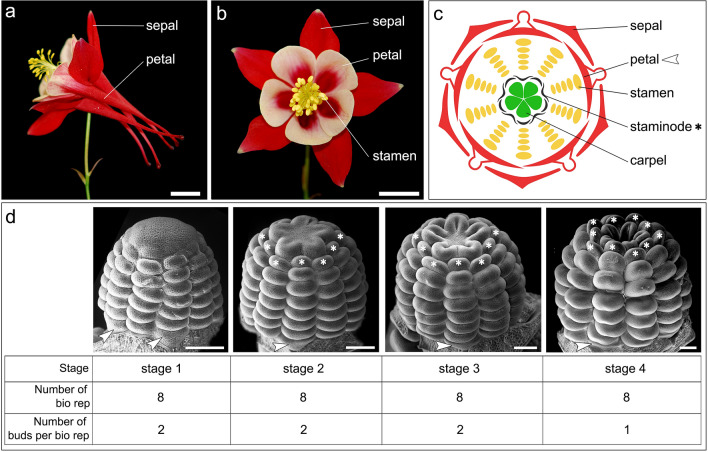
*A. coerulea* and the floral developmental stages used for RNA-seq. (**a**) Side view of a mature *A. coerulea* flower. (**b**) Front view of a mature *A. coerulea* flower. (**c**) Floral diagram of a typical *A. coerulea* flower. (**d**) Developmental stages, the number of biological replicates per stage, and the number of floral buds used for each biological replicate that were used for RNA-seq. Asterisks indicate staminodes and arrowheads indicate petals. Scale bar: a,b = 1 cm; d = 100 µm.

In this study, we have conducted the first in-depth transcriptional profiling of early floral development in *Aquilegia coerulea* at four finely-dissected stages (Fig. 1). The genus *Aquilegia* belongs to the basal eudicot buttercup family Ranunculaceae and is a model system for floral evolutionary developmental studies^8^. Analysis of floral ontogeny^9^ has shown that *Aquilegia* floral organ primordia initiate in whorls of five organs each, which are arranged in 10 orthostichies (vertical rows of organs), with alternate orthostichies either above the sepals or the petals (Fig. 1c). *Aquilegia* flowers all have multiple whorls of stamens, which is one of the major differences relative to other established model systems (e.g. *A. thaliana*, petunia, snapdragon) that all have only one whorl of stamens. The flowers of *A. coerulea*, as well as those of almost all other *Aquilegia* species, possess a fifth type of floral organ, the staminodes, which initiate in two whorls positioned between the stamens and carpels (Fig. 1). Unlike the syncarpous gynoecium of other model systems, *Aquilegia* is apocarpous with five distinct carpel primordia (Fig. 1). Using a candidate gene approach, previous studies have revealed the sub-functionalization of the B-class organ identity genes^10–12^ and that the *JAGGED* homolog is crucial for initiation of the floral organ primordia^13^, but genome scale studies to date have focused on late stage floral organ development^14–16^.

The current experiment was designed to obtain a broad characterization of the genetic pathways acting in early *Aquilegia* FM development. In order to ensure the power and accuracy of our sequencing results, we used eight biological replicates per developmental stage and obtained an average of 18× sequencing depth per sample. The developmental window we sequenced sampled stages that started with a late phase of stamen initiation, covered the period of FM termination, and ended with the initial stage of morphogenesis of the floral organs. Using differential expression (DE) analysis and weighted gene co-expression network analysis (WGCNA), we identified crucial genes marking the transitions between developmental stages, and hub genes in co-expression modules that are strongly associated with these developmental stages. Our results support the potential functional conservation of key genes in early floral development that have been identified in other systems, but also revealed a number of previously unknown or overlooked loci that are worthy of further investigation. In addition, our results highlight not only the dynamics of transcriptional regulation during early floral development, but also the potential involvement of the complex, essential networks of small RNA and post-translational regulation in this process.

## Materials and methods

### Plant materials: growing conditions and dissection

Seeds of *Aquilegia* x *coerulea* ‘Kiragami’ were purchased from Swallowtail Garden Seeds (Santa Rosa, CA, USA). Seeds were germinated in wet soil and seedlings were grown under conditions of 16 h daylight at 18 °C and 8 h dark at 13 °C. Once the plants developed approx. six true leaves, they were transferred into vernalization conditions (16 h daylight at 6 °C and 8 h dark at 6 °C) for three to 4 weeks, and subsequently moved back to the regular growth condition. Once the inflorescences started to develop, axillary FMs were quickly dissected on ice using surgical needles and flash frozen in liquid nitrogen. Three sepals were removed from every floral bud collected.

### Scanning electron microscopy

Floral buds were dissected using the same method as above, fixed in FAA (10% formaldehyde, 50% ethanol, 5% acetic acid), and stored at 4 °C. Prior to imaging, samples were dehydrated through a graded ethanol series to 100% and then critical point dried with CO_2_ (Autosamdri-815, Tousimis, MD, USA). Images were taken with the JSM-6010 LC Scanning Electron Microscope (JEOL, MA, USA) at the Arnold Arboretum of Harvard University.

### RNA extraction, library construction, and sequencing

The total RNA of all samples was extracted using the RNeasy Micro Kit (Qiagen, Netherlands). The integrity of extracted RNA was measured by using a 2200 TapeStation (Aligent Technologies, CA, USA). Subsequently, each RNA sample was diluted to a concentration of 1.25 ng/µl, measured by using Qubit RNA HS Assay Kit (Thermo Fisher Scientific, MA, USA). Sequencing libraries were prepared by the Bauer Core facility of Harvard University using the SMART-Seq v4 Ultra Low Input RNA Kit (Takara Bio USA, Inc., CA, USA) for cDNA synthesis and Illumina Nextera XT (Illumina) for library preparation. Libraries were fragmented to an average size of 350 bp. Library concentrations were examined using a Qubit 2.0 (Thermo Fisher Scientific, MA, USA) and qRT-PCR, and the insert sizes measured using a Bioanalyzer 2100 (Aligent Technologies, CA, USA). Libraries were then pooled and diluted to a final concentration of 3 nmol. Sequencing was conducted at Novogene (Beijing, China) using the 150 bp paired-end Illumina HiSeq4000 sequencing platform.

### Mapping reads to the reference transcriptome

The raw sequencing reads were filtered by removing adapters and low-quality reads using Trim Galored v0.6.5^17^ (quality Phred score cutoff = 20) and mapped to the *Aquilegia* × *coerulea* ‘Goldsmith’ v3.1 reference transcriptome (https://phytozome.jgi.doe.gov/) using Kallisto v0.46.1^18^. Raw and processed reads are deposited under the GEO accession number GSE158507.

### Differential expression (DE) analysis

Read counts and transcript per million reads (TPMs) were generated using the R package tximport v1.0.3 and the lengthScaledTPM method^19^. Lowly expressed transcripts were filtered based on analyzing the mean–variance trend, and transcripts with more than 1 counts per million reads in at least one of the 32 samples were retained. Reads were normalized using the variance stabilizing transformation (VST) method to perform principal component analysis (PCA). Differential gene expression analysis was conducted following the DESeq2 R package v1.28.1^20^. For DE genes, the log2 fold change of gene abundance was calculated between pairwise groups, and the significance of expression changes was determined using the Student’s *t-*test. *p* values were adjusted with the Benjamini–Hochberg Procedure to correct for the false discovery rate^21^. A gene was considered to be significant if it had adjusted *p* value < 0.05 and log2 fold change ≥ 1. A heatmap of all DE genes was produced using the R package ComplexHeatmap v2.2.0. Hierarchical clustering was used to partition the DE genes into two clusters with Euclidean distance and ward.D2 clustering algorithm.

### Gene ontogeny (GO) enrichment and kyoto encyclopedia of genes and genomes (KEGG) pathway analysis

Gene Ontogeny (GO) analysis for DE groups and WGCNA modules of interest was calculated using agriGO v2 (https://systemsbiology.cau.edu.cn/agriGOv2/)^22,23^ and the KEGG pathway analysis^24,25^ was performed using the DAVID Bioinformatics Resources 6.8 (https://david.ncifcrf.gov/)^26,27^. For both analyses, the identifiers of the top-hit *A. thaliana* loci were obtained from the *A. coerulea* “Goldsmith” v3.1 reference genome annotation (https://phytozome.jgi.doe.gov/), all of the expressed genes in the *Aquilegia* RNA-seq data were used as the background reference. Enrichment in GO terms or pathways was calculated with Fisher’s exact test, and *p* values were adjusted for the false discovery rate using the Benjamini–Hochberg procedure. The minimum number of mapping entries for a GO category was set to be 5, and the count threshold for KEGG pathway analysis was set to be 2. Enrichment figures were produced using the R package ggplot2.

### WGCNA

Weighted gene correlation network analysis (WGCNA)^28^ was constructed using the R package WGCNA v1.68 following the package tutorials; the illustrated workflow is shown in Fig. S1. To avoid noise from lowly expressed genes, all genes that had read counts less than 10 in more than 90% of the samples (i.e. 29 samples) were removed. The expression of the resultant 18,303 genes was then normalized using the varianceStabilizingTransformation function from the R package DESeq2^20^. The adjacency matrix was calculated using a soft thresholding power of 5. Compared to a hard threshold, of which the correlation values above or below are considered to be connected or not, respectively; the soft threshold is used to raise the correlation to a power so that the difference between strong and weak correlations are exaggerated rather than defined into binary terms. The topological overlap matrix was calculated using a deepSplit value of 4, minModuleSize was set to 20, and mergeCutHeight was set to 0.2. Node and edge information of modules of interest were exported using the exportNetworkToCytoscape function of the WGCNA package. The node and edge files were then transformed into .json format using the R package jsonlite v1.6.1, and visualized using customized scripts modified based on the D3 JavaScript library.

### Gene phylogenies

Neighbor-joining phylogenetic trees for genes that are discussed in the main text were constructed if the homologs in *A. coerulea* and *A. thaliana* were not each other’s reciprocal top BLAST hits. Potential homologs of the genes of interest were identified from the genomes of diverse species on Phytozome, all amino acid sequences were aligned using ClustalW^29^ and the neighbor-joining trees were constructed using MacVector v17.5.5(Gary, NC, USA).

## Results and discussion

### Developmental stages and sequencing information for transcriptome profiling

In order to finely dissect and capture the transcriptional dynamics during early phases of *Aquilegia* floral development, we defined four developmental stages for RNA sequencing (Fig. 1d). At stage 1 (s1), the dome of the FM is round and visible, and stamen primordia are in the progress of initiating. At stage 2 (s2), all the stamen and staminode primordia have completed initiation, the five carpel primordia are about to initiate, and the apex of the floral bud is star-shaped and flattened. At stage 3 (s3), the outer rim of each carpel primordium is elevated but the development of carpel primordia has not consumed all the cells of the apex, and the stamen and staminode primordia are still morphologically indistinguishable. At stage 4 (s4), the carpel primordia continue to develop and elongate, all the cells in the apex have been consumed by carpel development, and the staminode primordia have started to expand laterally, making them morphologically distinct from the stamen primordia.

A total of 72 floral buds were dissected from 30 individual plants for RNA extraction. For s1, s2, and s3, each biological replicate contained two floral buds from two individuals, while s4 contained one floral bud per biological replicate (Fig. 1d). The amount of total RNA per extraction varied from 20.2 to 138 ng. After quality assessment, eight samples at each stage with the best RNA qualities were used for subsequent library construction. All 32 selected samples had RNA integrity numbers higher than 8.9.

**Figure 2:**
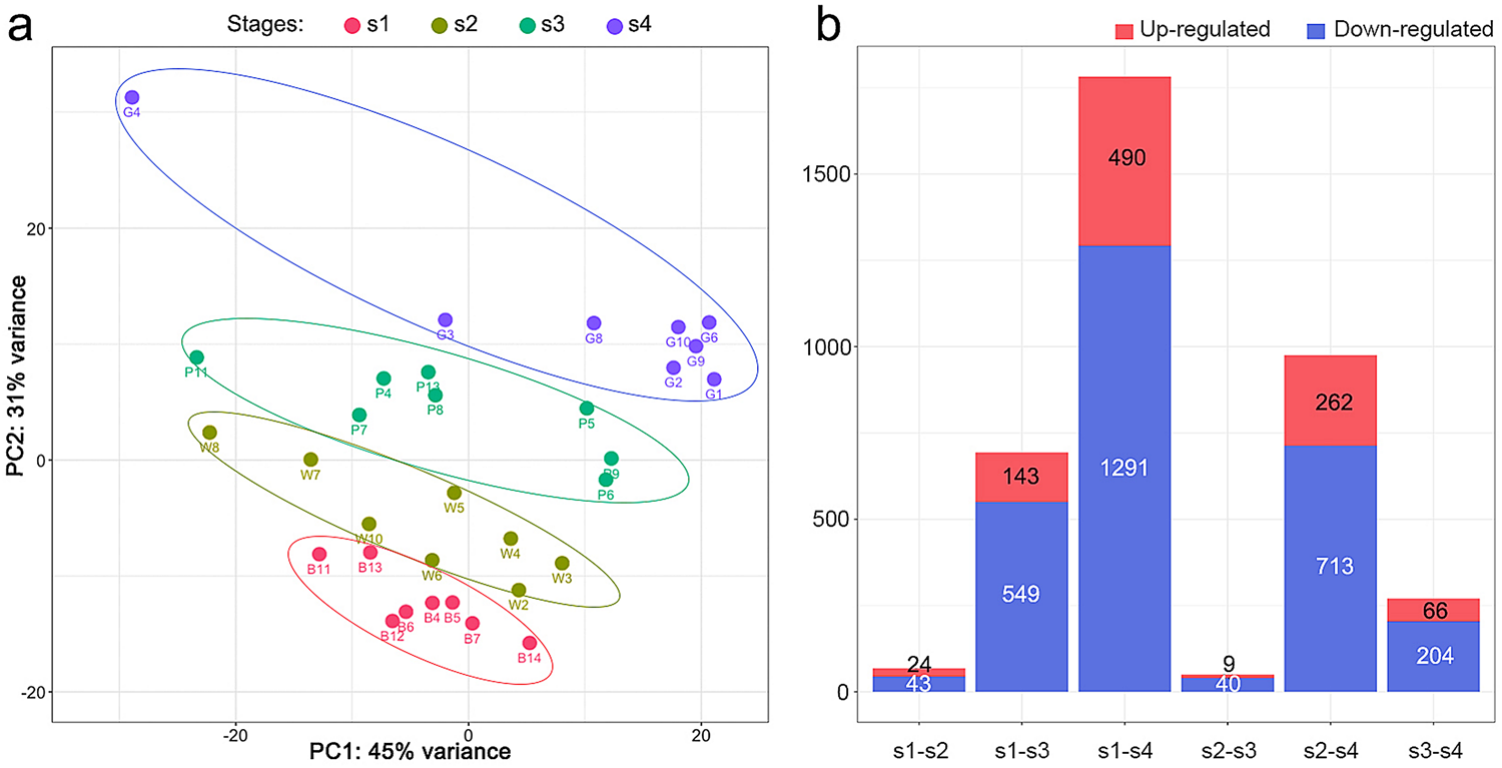
PCA of normalized reads of all samples (**a**) and bar plot summary of DE genes between developmental stages (**b**).

A total of 993.64 million raw reads (323.1G raw data) were generated from the 150 bp paired-end Illumina HiSeq4000 sequencing platform. The number of reads generated per sample ranged between 25 and 36 million (Fig. S2), and the average reads per developmental stage ranged from 29.3 to 32.9 million (Fig. S2). After filtering reads by quality (Phred score cutoff = 20), the fraction of reads retained ranged from 80.2 to 88.4% (Fig. S2). Retained reads were then mapped to the *Aquilegia* × *coerulea* ‘Goldsmith’ v3.1 reference transcriptome, generating on average 17.6× to 19.8× sequencing coverage per developmental stage (Fig. S2), and a total of 30,023 raw transcripts were mapped. Subsequently, lowly expressed transcripts were filtered if a transcript had less than 1 million read count in more than 31 samples, which resulted in 20,473 expressed genes in all samples. To conduct a preliminary exploration of the 32 samples, normalized reads of all samples were used to perform a principal component analysis (PCA; Fig. 2a). The two primary PCs explained 76% of the total variance among all samples and a clear clustering by developmental stages was observed (Fig. 2a).

### Differential expression analysis between developmental stages

To identify genes that were significantly differentially expressed (DE) between developmental stages, the log2 fold change of the gene abundance was calculated between different stages. A gene was considered DE if it had an adjusted *p* value of < 0.05 and an absolute value of log2 ratio $\ge$ 1. A total of 1944 DE genes were identified, with more genes upregulated at s4 compared to any other developmental stage (Fig. S3; Supp. Data 1). For each pairwise comparison, there were consistently more genes down-regulated at the earlier stage relative to the later stage (Fig. 2b; Supp. Data 1).

A small number of DE genes, 67 and 49, were identified when comparing s1 to s2, and s2 to s3, respectively. Among these 67 and 49 DE genes, only five loci (Table S1) appeared in both comparisons, suggesting that although s2 appeared to be transcriptionally similar to both s1 and s3, the similarities lay in different aspects in each comparison. One of the five genes in common, *Aqcoe7G234000*, is the homolog to the *ARABIDOPSIS HISTIDINE PHOSPHOTRANSFER PROTEIN 6* (*AHP6*). It is also the top DE gene that is significantly up-regulated in s1 compared to s2, and among the top DE genes that are up-regulated at s2 compared to s3 (Table S1). In *A. thaliana, AHP6* is one of the key genes mediating crosstalk between the auxin and cytokinin signaling pathways, and participates in a number of crucial developmental processes, including specifying the founder cells for lateral roots, protoxylem, and protocambium^30^. In shoot apical meristems (SAMs), inflorescence meristems (IMs), and FMs, *AHP6* is involved in the patterning of phyllotaxy and organ initiation^30–32^. In particular, the AHP6 protein functions non-cell-autonomously to create a cytokinin-signaling inhibition field that contributes to the robustness of the auxin-signaling inhibition field for phyllotaxy and organ patterning^31^. We hypothesize that the *AHP6* homolog in *Aquilegia* is functionally conserved since the expression level of *AqAHP6* sharply declines as organ initiation ceases during the progression of s1 to s4 (Fig. S4).

Among the 24 genes that were found to be significantly up-regulated at s1 relative to s2, *Aqcoe3G399500* is the top DE gene after *AqAHP6* (Table S1). The *A. thaliana* homolog of this locus is *PERIANTHIA* (*PAN*), which has been shown to regulate floral architecture and directly activate the expression of the C-class gene *AGAMOUS* (*AG*)^33^. Homologs of other genes that specify meristem and organ primordia boundaries during early floral development also appeared to be significantly up-regulated at s1, including *CUP SHAPED COTYLEDON3*^34^ (*AqCUC3*; *Aqcoe6G165500*) and *HANABA TARANU*^35^ (*AqHAN*; *Aqcoe5G190300*), as well as loci that are known to regulate floral organ size and growth, such as *STERILE APETALA*^36^ (*AqSAP*; *Aqcoe1G384300*) and *AINTEGUMENTA-like 6*^37^ (*ATL6/PLT3*; *Aqcoe6G092100*).

Interestingly, two key genes controlling stomata development in various plant lineages, *AqMUTE* and *AqEPF1*^38,39^, were both expressed at very low levels at s1, but significantly up-regulated in s2 followed by consistent high expression at later stages (Fig. S4). This may suggest that although s1 sepals have already achieved reasonable sizes, stomatal development does not initiate until s2. Other genes that were shown to be up-regulated in s2 compared to s1 include the adaxial identity gene *AqCRC*, which is consistent with previous studies showing that strong *AqCRC* expression coincident with the initiation of the carpel primordia^15^.

The top DE gene that is up-regulated in s2 relative to s3, *Aqcoe7G055500*, encodes a non-specific serine-threonine kinase with a predicted RNA-binding domain, which does not appear to be a member of any of the better-known serine-threonine classes^40^. Its homolog in *A. thaliana*, *AT5G51800*, has not been studied functionally but is highly expressed in the shoot apex, inflorescence meristem, developing carpels, and stigma (Arabidopsis eFP Browser 2.0). However, this gene is highly expressed at s2 in the current study, in which the FM has just completed organ primordia initiation, and it will, therefore, be interesting to examine its function in early *Aquilegia* floral development.

**Figure 3:**
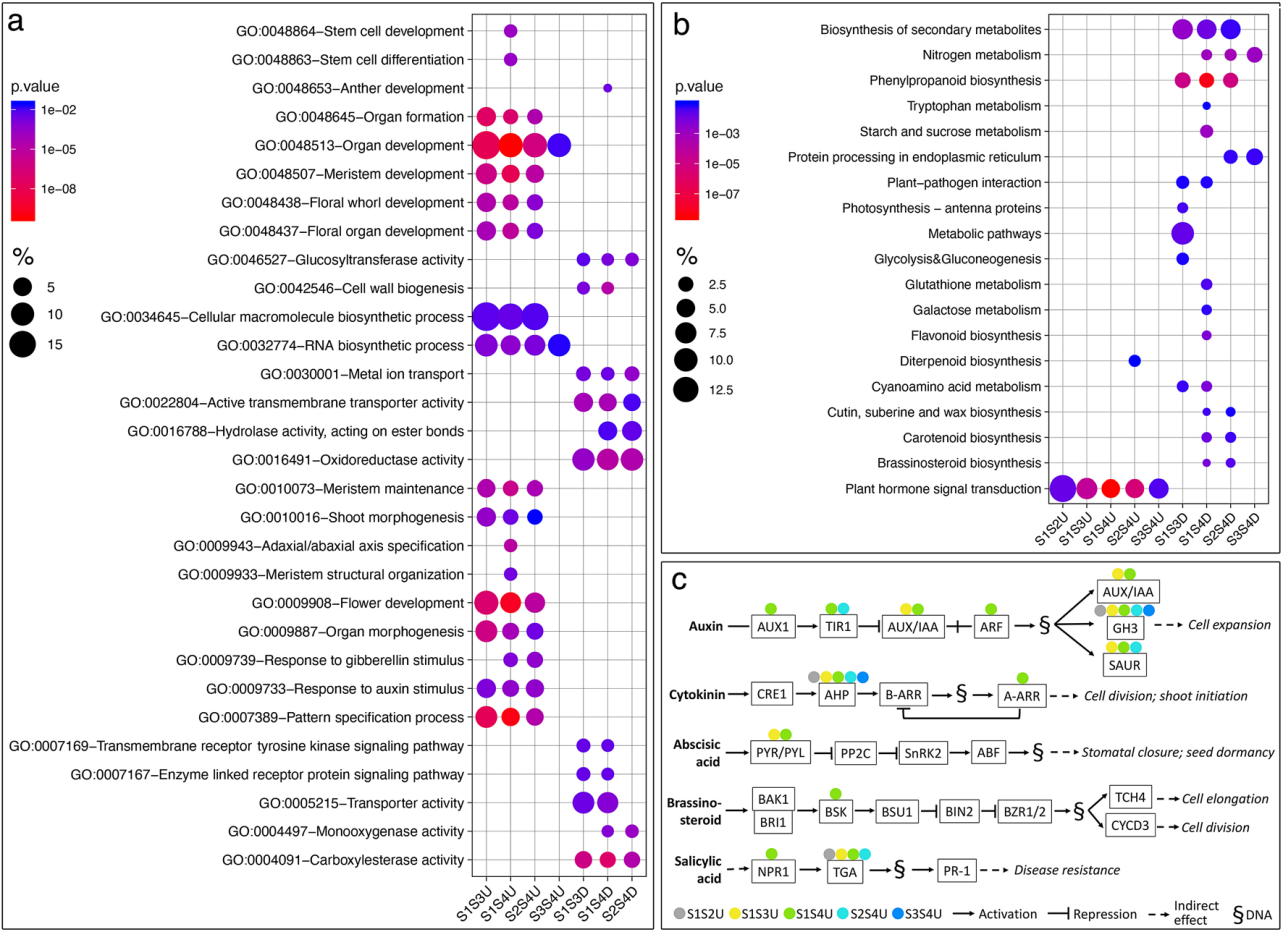
GO term and KEGG pathway enrichment analysis of DE genes. (**a**) Selected enriched GO terms from different DE comparisons. (**b**) Selected enriched KEGG pathways from different DE comparisons. (**c**) Enriched plant hormone signal transduction pathways from different DE comparisons. % in a and b were calculated as: number of genes that hit the specific GO term or KEGG pathway/total number of genes in the input DE list*100. Each DE comparison is noted as: earlier stage vs. later stage, up- (U) or down (D)-regulated at the earlier stage.; for instance: S1S2U means genes that are DE between s1 and s2 and are up-regulated at s1.

Gene ontogeny (GO) analysis of DE genes revealed a wide range of enrichment terms for every DE comparison except for [s1 vs. s2] and [s2 vs. s3], likely due to the small numbers of DE genes in these two comparisons, as mentioned above (Supp. Data 2). In general, there seems to be a large portion of overlap in GO terms in DE genes that were up- or down-regulated during early stages compared to later stages (Fig. 3a). For instance, GO terms that are related to organ formation, patterning, and development are enriched for DE genes that are up-regulated at s1 and s2. On the other hand, genes that are up-regulated at s3 and s4 are heavily enriched in metabolic and enzymatic activities, including active transmembrane transportation, carboxylesterase activity, oxidoreductase activity, and glucosyltransferase activity, all of which are involved in broader metabolic processes during plant development.

Subsequently, we conducted KEGG pathway enrichment analysis with the DE genes, and we observed a similar pattern for genes that are up-regulated in s4, in that they participate in various metabolic pathways (Fig. 3b). For instance, genes involved in nitrogen metabolic pathways are up-regulated and enriched in s4, indicating active amino acid biosynthesis and protein synthesis at s4 compared to earlier developmental stages. On the other hand, all the genes that are up-regulated at the earlier stages in each DE comparison are almost exclusively enriched in plant hormone signal transduction pathways (Fig. 3b).

We then examined the hormonal signaling pathways that were enriched for each DE list, which revealed several interesting points (Fig. 3c). Firstly, all DE lists contained genes in the auxin signaling pathways. The DE genes up-regulated in s1 compared to s4 included genes in all major components in auxin signaling, while all other DE lists only showed enrichment in a few components in the pathway. This indicates a gradual decrease in the number of highly expressed auxin signaling genes from s1 to s4. Secondly, as mentioned previously, *AqAHP6* appeared to be differentially expressed at the earlier stages in all DE comparisons, which was also captured by the KEGG pathway analysis as enriched in AHP family members. Besides *AqAHP6*, homologs of the Arabidopsis Response Regulator (ARRs) proteins were shown to be up-regulated in s1 compared to s4, and ARRs are downstream of AHPs in the cytokinin signaling pathway, which emphasizes the decrease in cytokinin signaling from s1 to s4. Thirdly, genes that are up-regulated at s1 compared to s4 are detected from the abscisic acid, brassinosteroid, and salicylic acid signaling pathways, suggesting a complex interplay between multiple plant hormone pathways during the early stages of floral development.

**Figure 4:**
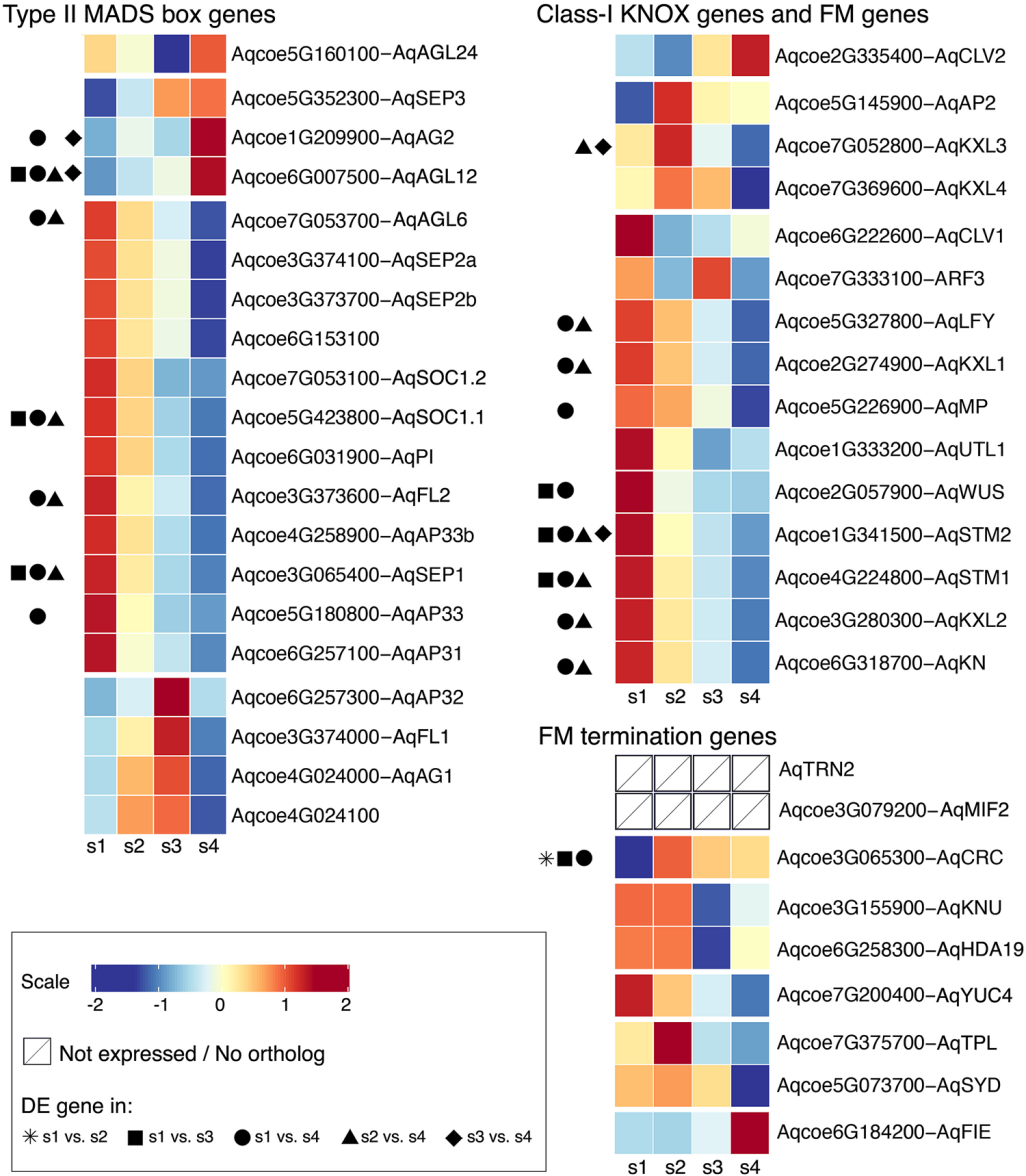
Expression profiles of type-II MADS-box genes, class-I KNOX genes, and genes involved in FM maintenance and termination pathways. Expression levels of each gene over the developmental stages were scaled as the average expression of all biological replicates per stage subtracted by the average expression of all replicates of all stages, then divided by the standard deviation of the expression of all replicates of all stages. DE genes were indicated with symbols of each DE comparison.

We profiled the expression of type-II MADS-box genes^41,42^ and class-I KNOTTED-like homeodomain (KNOX) genes^14^, because many members of these families are known to play important roles during early floral development, along with additional homologs of genes involved in FM identity and maintenance (Fig. 4). Among the type-II MADS-box genes, the C-class gene *AqAG2* and *AGAMOUS-LIKE 12* (*AqAGL12*) showed a significant increase in the expression levels from s1 to s4. This might be expected for *AqAG2*, which is known to be strongly expressed in carpels^10^, but expression of *AqAGL12* has not previously been investigated in *Aquilegia*. While the majority of the MADS-box genes showed a decrease in their expression levels from s1 to s4, this trend was only significant for *SUPPRESSOR OF OVEREXPRESSION OF CONSTANS 1* (*AqSOC1.1*), *FRUITFULL-LIKE 2* (*AqFL2*), *SEPALLATA1* (*AqSEP1*) and *APETALA3-3* (*AqAP3-3*) (Fig. 4). Similarly, most of the class-I KNOX genes and FM-related genes showed the highest expression levels at s1 with a decrease at subsequent stages, but *AqKXL3* appeared to be expressed at the highest levels at s2 (Fig. 4).

Since the meristem stages we sampled captured the transition from organ primordia initiation to FM termination (i.e. the end of organogenesis), we also examined the expression of the homologs of genes in the known FM termination pathways^4,5,43–46^ (Fig. 4). Interestingly, several lines of evidence suggest limited conservation of these pathways, particularly the *KNU*-*WUS* pathway, between *A. thaliana* and *A. coerulea*. In *A. thaliana, KNU* has been shown to be essential in terminating the expression of *WUS* in the FM, and continuous expression of *KNU* appeared to be necessary to maintain the suppression of *WUS* via both transcriptional suppression and heterochromatinization^44^, but the expression of *AqKNU* (Figs. S4 and S6) decreased after s2. Furthermore, *KNU* and *SPLAYED* (*SYD*) function antagonistically by competing for a binding site in *WUS* promoter, but *AqKNU* and *AqSYD* showed similar, rather than the opposite, patterns of expression over s1 to s4 (Fig. S4). Additionally, FERTILIZATION-INDEPENDENT ENDOSPERM (FIE) is a key polycomb repressive complex2 component, which has been shown to physically interact with KNU to deposit repressive H3K27me3 marks and maintain the stable silencing of *WUS* in later developmental stages^44^. Unlike what was observed in silencing *FIE* expression in *A. thaliana*, however, a previous study has shown that the silencing of *AqFIE* did not lead to indeterminacy in the flowers^47^. MINI ZINC FINGER2 (MIF2) was shown to be a component of a protein complex together with KNUCKLES (KNU), TOPLESS (TPL) and HISTONE DEACETYLASE-like 19 (HDA19) to suppress the expression of *WUS* in the FM^43^, but the read counts of the *MINI ZINC FINGER2* (*MIF2*) homolog were extremely low in all sample of all stages and this gene is considered as not expressed in our dataset. Besides the *KNU-WUS* pathway, *TORNADO2* (*TRN2*) functions downstream of *CRC* in coordinating FM termination and carpel primordia formation in *A. thaliana*^46^, but phylogenetic analysis did not recover any ortholog of *TRN2* the *Aquilegia* genome (Fig. S5). These findings suggest that the *A. thaliana* mechanisms controlling FM termination are not well conserved in *Aquilegia*.

### Gene co-expression analysis, identification of module hub genes, and network construction of modules of interest

**Figure 5:**
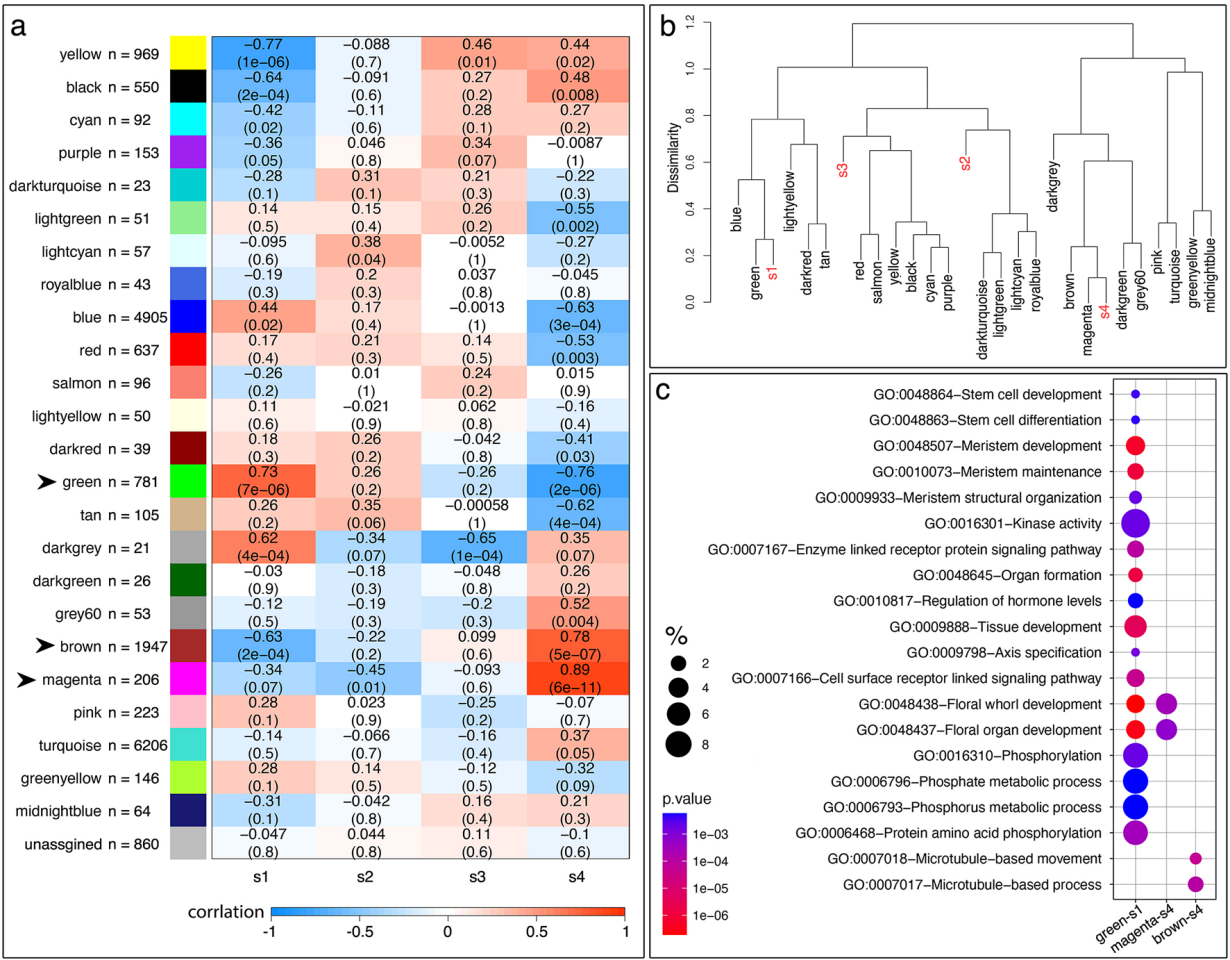
Associating gene co-expression modules with developmental stages. (**a**) Correlation between module eigengene values and the developmental stages. The first number in each cell represents the correlation value, and the second number in the parenthesis represents the p-value of the correlation. Arrowheads pointed to modules of interest. (**b**) Hierarchical clustering dendrogram of the eigengenes of modules and the developmental stages. (**c**) Selected GO term enriched from modules of interest. % was calculated as: number of genes hit the specific GO term/total number of genes in the input list*100.

The DE analysis provided us with a good starting point of what genes might be functionally relevant to a certain developmental stage based on the changes in their expression levels, but a strong limitation for DE analysis is that each gene is considered in isolation while in reality, genes and gene products function in networks. In order to discover co-expressed genetic modules that are significantly associated with different stages during early floral development in *Aquilegia*, we conducted weight gene co-expression network analysis (WGCNA) using the RNA-seq data (refer to Fig. S1 for an illustrated workflow). Using a soft threshold power = 5 (Fig. S7), a total of 24 co-expression modules were constructed from 18,303 genes, with the smallest module (darkgrey) containing 21 genes and the largest module (turquoise) containing 6206 genes (Fig. 5a). Subsequently, we looked at the association between the modules and the four developmental stages by correlating the eigengene value (i.e. a value equivalent to the first component of the module; a singular value decomposition to summarize the expression levels of all genes in that module) of the modules with the stages. Among the 24 modules, we determined three modules to be of particular interest based on their correlation (Fig. 5a) and close distance in the hierarchical dendrogram clustering (Fig. 5b): the green module for its strong association with s1, and the brown and magenta modules for their strong association with s4. We will refer to the modules as green-s1, brown-s4, and magenta-s4 hereafter for clarity.

To obtain a broad overview of genes in the three modules of interest, we performed GO and KEGG pathway analysis on these modules (Fig. 5c; Supp. Data 2). GO analysis revealed that although the green-s1 module has a moderate size (781 genes) compared to other modules, it has the largest number of enriched GO terms, most of which are specific to early floral developmental stages, such as stem cell/meristem maintenance and development, axis specification, and organ formation (Fig. 5c; Supp. Data 2). On the other hand, the brown-s4 module has a relatively large number of genes (1947 genes) but only two highly related cellular process GO terms are found to be significant, microtubule-based processes and movement (Fig. 5c; Supp. Data 2). The magenta-s4 module also shows strong and significant association with s4, but genes in this module are enriched in “floral whorl” and “floral organ development”, particularly in androecium, perianth, and gynoecium development (Fig. 5c; Supp. Data 2). This suggests that the WGCNA has identified modules that are significantly associated with different aspects of floral development at s4: genes in the brown-s4 module appear to be focused on the rapid growth of all floral organs in s4, in which active mobilization of the microtubules is necessary for rapid cell expansion; while genes in the magenta-s4 module are specific to the elaboration and maturation of each floral organ identity.

KEGG analysis revealed that the green-s1 and brown-s4 modules both contain genes that are involved in the starch and sucrose metabolism pathways (Supp. Data 2). In addition, genes in the green-s1 module are significantly enriched in the plant hormone signal transduction pathways, and genes in the brown-s4 module are significantly enriched in several sugar metabolic pathways, including fructose and mannose metabolism, and amino sugar and nucleotide sugar metabolism (Supp. Data 2). Sugar metabolism has been implicated in various aspects of plant development, especially the amino sugar and nucleotide sugar interconversion pathways that are required for cell wall biosynthesis^48^. Together with the GO enrichment in microtubule-based movement and processes for module brown-s4, this again emphasizes the initiation of rapid organ growth during s4 of floral development in *Aquilegia*. No pathway enrichment was found for genes in the magenta-s4 module, possibly due to the small module size.

**Figure 6:**
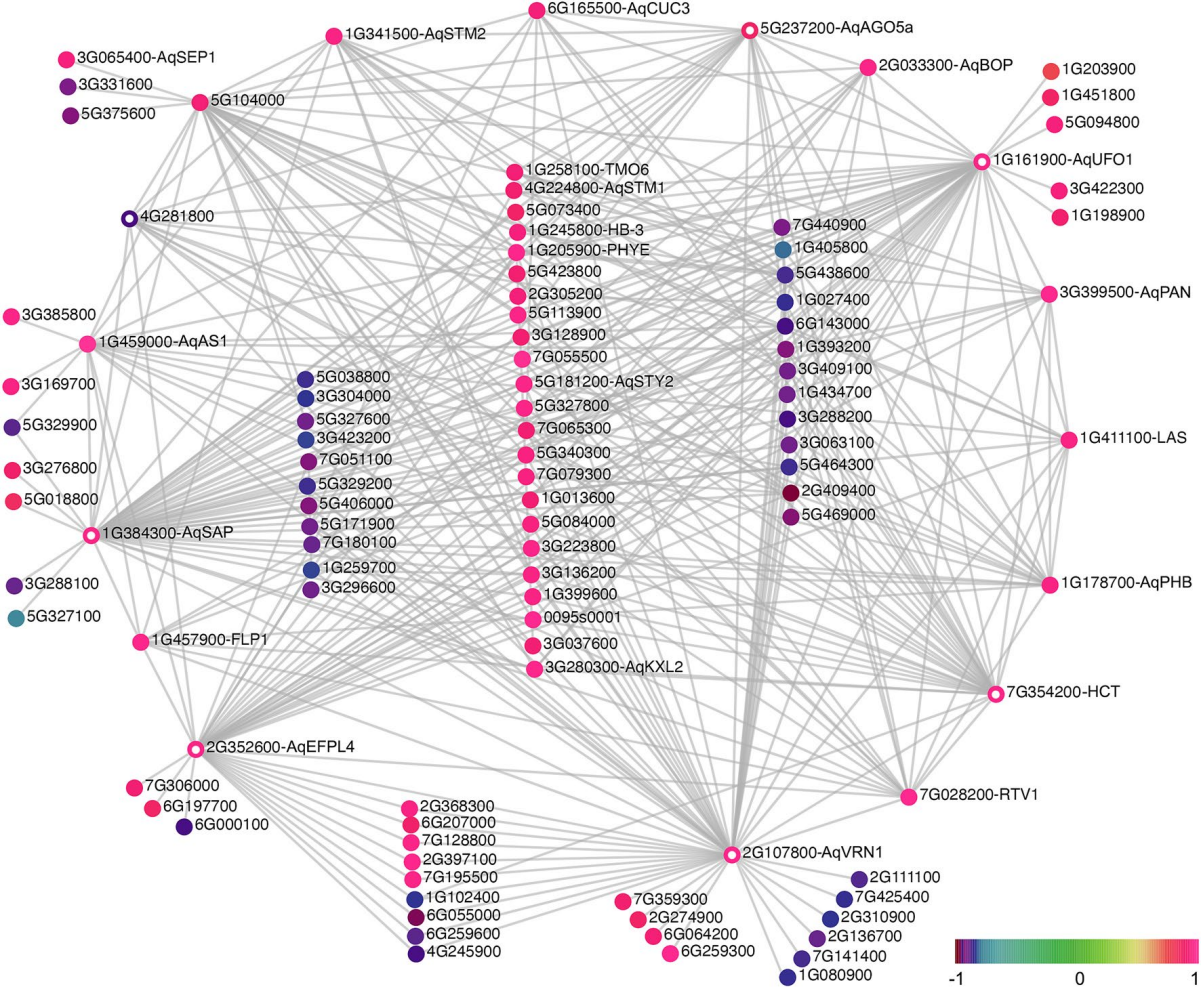
Gene network of module green-s1. Color scale represents the value of MM for each gene. The “Aqcoe” prefix of a gene identifier was removed to facilitate the visualization. All the genes with the largest number of connections are at the periphery of the network (i.e., genes in the center have fewer connections compared to genes at the periphery). Genes that are presented as colored circles (instead of a solid dot) are genes that were discussed in the main text.

Subsequently, we identified the potential hub genes of each module of interest based on the value of signed module membership (MM) and trait significance (TS). The former describes the correlation between a gene and the eigengene value of a module, while the latter describes the correlation between a gene and the trait of interest; the potential hub genes of a module should have significant and strong correlations with both MM and TS (Figs. S1 and S8). We, therefore, defined the hub genes as having their absolute values of MM and TS above the 90th percentile of the distribution of MM and TS of all the genes of the module. This approach identified 33, 56, and 14 hub genes for the green-s1, brown-s4, and magenta-s4 modules, respectively (Fig. 6; Table S2). Moreover, although every gene is connected to all other genes in a module, the strengths of the connection between gene pairs vary and the hub genes, by definition, should have the strongest values of connectivity. To better visualize the gene co-expression networks of each module, we only retained the edges that have the highest weight values (i.e. having the strongest connectivity) with the hub genes in the network for visualization (Figs. S8, 6, 7 and 8). To facilitate navigation among a group of highly interconnected genes (particularly in the case of the green-s1 and brown-s4 modules), we moved all the genes with the largest numbers of connections to the periphery of the visualized network and thus genes left in the center of the network had fewer connections compared to those of at the periphery.

**Figure 7:**
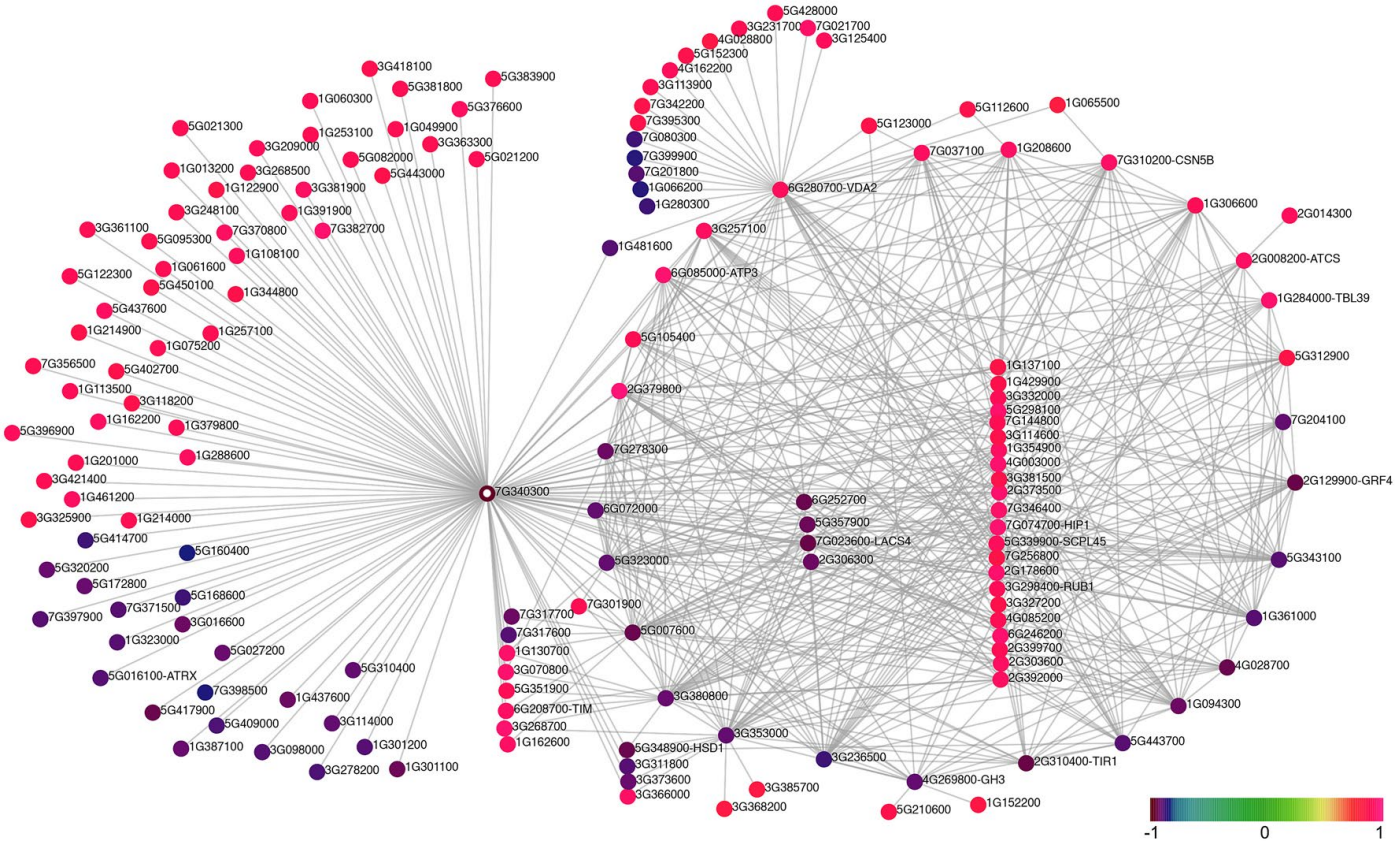
Gene network of module brown-s4. Color scale represents the value of MM for each gene. The “Aqcoe” prefix of a gene identifier was removed to facilitate the visualization. All the genes with the largest number of connections are at the periphery of the network (i.e. genes in the center have fewer connections compared to genes at the periphery). Genes that are presented as colored circles (instead of a solid dot) are genes that were discussed in the main text.

**Figure 8:**
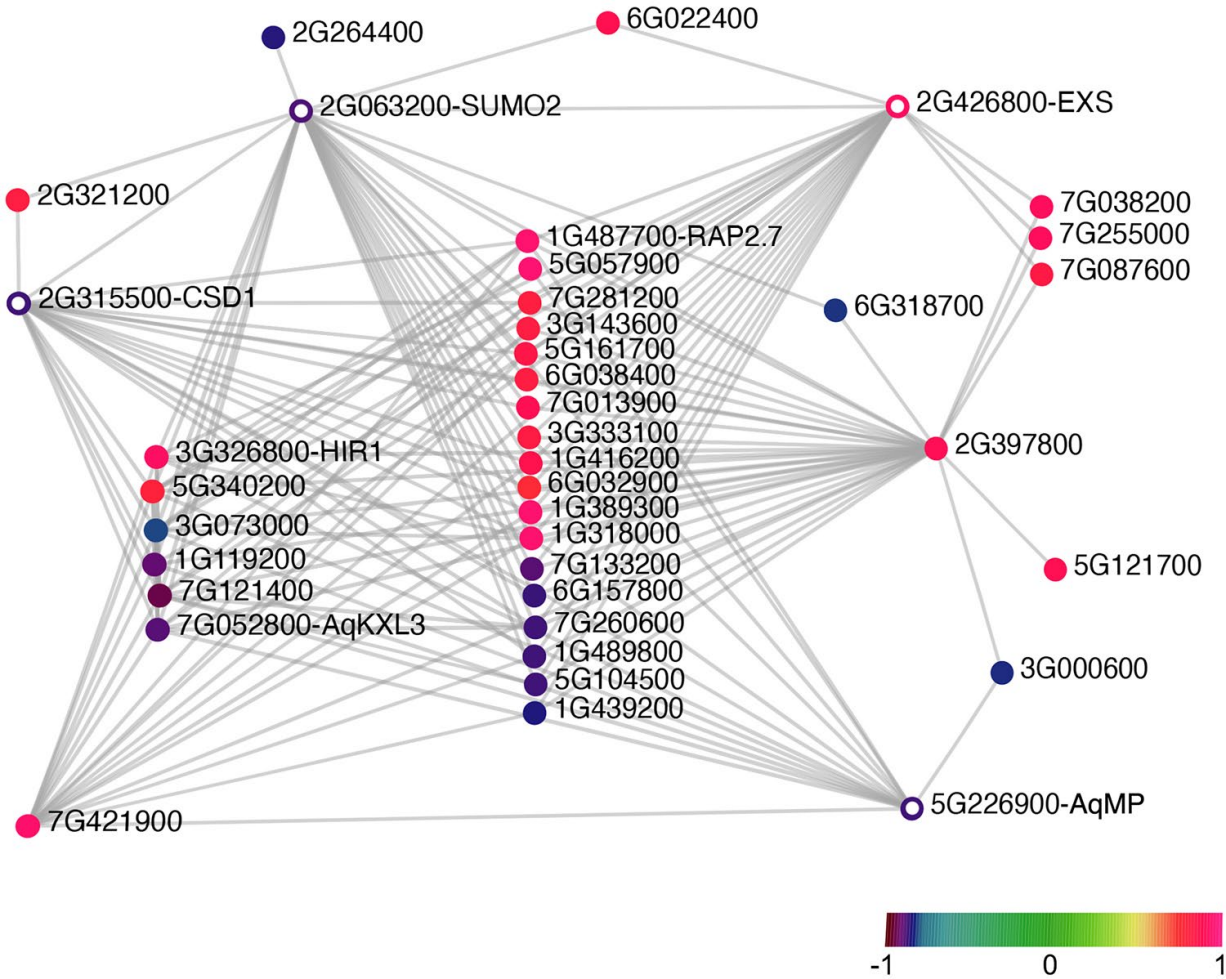
Gene network of module magenta-s4. Color scale represents the value of MM for each gene. The “Aqcoe” prefix of a gene identifier was removed to facilitate the visualization. Genes that are presented as colored circles (instead of a solid dot) are genes that were discussed in the main text.

### Hub genes of the green module revealed new candidate genes associated with s1 floral development

The green-s1 module contains 33 potential hub genes and 17 of these encode transcription factors (TFs). The homologs of many of these TF-coding genes are involved in early FM and floral development, such as class-I KNOX genes (*AqSTM1*, *AqSTM2*, *AqKXL2*), *AqAS1*, *AqSEP1*, *AqPAN*, and the homologs of *CUP SHAPED COTYLEDON3* (*Aqcoe6G165500*; *AqCUC3*), *HOMEOBOX-3* (*Aqcoe1G245800*; *AqHB3*), *PHABULOSA* (*Aqcoe1G178700*; *AqPHB*), *LATERAL SUPPRESSOR* (*Aqcoe1G411100*; *AqLAS*), *BLADE ON PETIOLE2* (*Aqcoe2G033300*; *AqBOP*), and *STERILE APETALA* (*Aqcoe1G384300*; *AqSAP*). Of these, *AqPAN* also showed the strongest association with s1 (Table S2).

Interestingly, although all the potential hub genes have similarly high MM and TS values (Table S2), the number of edges connected to the hub genes in the network appeared to be highly variable (Fig. 6), indicating a hierarchy of connectedness even among the hub genes. For instance, we have identified nine hub genes that have a strong negative association with the module and the s1 developmental stage (Table S2), but all of them except one (*Aqcoe4G281800*) have only a few edges connecting them with other genes in the network (and thus were not placed in the periphery of the network). *Aqcoe4G281800* encodes a wax synthase that is involved in constructing cell walls, and also only has a moderate number of edges compared to the those associated with other periphery hub genes. By contrast, the five periphery hub genes with the largest number of edges are (Fig. 6): *AqVRN1* (*Aqcoe2G107800*, homologous to *VERNALIZATION1*, *VRN1*, in *A. thaliana*); *AqHCT* (*Aqcoe7G354200*, codes for a hydroxycinnamoyl-Coenzyme A shikimate/quinate hydroxycinnamoyltransferase, HCT); *AqUFO1*(*Aqcoe1G161900*; *UNUSUAL FLORAL ORGANS1*^12^), *AqSAP;* and *AqEPFL4* (*Aqcoe2G352600*, homologous to *EPIDERMAL PATTERNING FACTOR-LIKE4*, *EPFL4*, in *A. thaliana*). The functional implications of some of these loci are unclear. For instance, *A. thaliana VRN1* belongs to the AP2/B3-like transcriptional factor family, and its function is primarily understood to be the regulation of the vernalization locus *FLOWERING LOCUS C* (*FLC*)^49^. However, although *Aquilegia* does require vernalization to flower^50^, it lacks an *FLC* homolog^51^, and *AqVRN1* is expressed in the early FM (Fig. S4), which differs considerably from the broad expression of *AtVRN1* in various organs and developmental stages of *A. thaliana*^49,52^. The potential role for *AqHCT* is equally enigmatic, as the enzyme appears to function in phenylpropanoid biosynthesis, a pathway that supports highly diverse cellular functions^53^.

Luckily, the remaining hub genes are better understood. Homologs of *UFO* encode F-box proteins that generally function with *LEAFY* (*LFY*) homologs to promote FM identity and activate B-class gene expression across diverse dicot model systems^54–58^. In *Aquilegia*, the closely related paralogs *AqUFO1* and *AqUFO2* promote the transition from inflorescence to FM identity in conjunction with *AqLFY*, although there is no clear role in B gene activation^12^. In addition to the expected contribution to FM identity, the *AqUFO1/2* paralogs also appear to promote the initiation of the stamen whorls, as whorl number dramatically decreases in silenced flowers from the usual 7–8 whorls to only 4–5. This function is intriguing for several reasons. First, the phenotype is only observed in *AqUFO1/2*-silenced flowers and not with *AqLFY*-silencing, suggesting that this function is specific to the F-box proteins and not a joint function with *AqLFY*. Second, it underscores a critical difference in *Aquilegia* floral development relative to other model systems in that, like many angiosperm flowers, *Aquilegia* has multiple whorls of stamens. The identification of *AqUFO1* as a critical hub gene in early floral development is consistent with this novel role in promoting stamen initiation and/or continued FM proliferation and points to other loci that may function as novel co-factors for this role.

In *A. thaliana*, *SAP* plays a number of roles, including acting as a cadastral gene that prevents *AG* from entering the perianth whorls, and controlling both inflorescence architecture and ovule development^59^. Interestingly, similar to UFO, SAP also contains an F-box motif and was recently shown to be a component of an Skp1-cullin 1-F-box (SCF) E3 ubiquitin-protein ligase complex that controls lateral organ size by promoting cell proliferation, a function that has been demonstrated both in *A. thaliana*^60^ and cucumber^61^. To date, the only genes that have been identified as the direct targets of the SAP-mediated E3 ligase complex are *PEAPOD1*/*2* (*PPD1*/*2*), which control organ growth^36,60^. The homolog of *PPD1* and *PPD2* in *Aquilegia*, *Aqcoe5G048100*, showed relatively stable expression from s1 to s4 (Fig. S4) and was assigned to the turquoise module (Fig. 5). It will be worth investigating whether this interaction is conserved in *Aquilegia* and what may be the other targets of the ligase complex during early floral development.

*AqEFPL4* belongs to the EPFL gene family that encodes small, secreted cysteine-rich ligands. The best-studied members of the family are the *EPIDERMAL PATTERNING FACTORS1*/*2*, which are key regulators in stomata development^38^. As mentioned above, *AqEPF1* was identified as one of the DE genes that is up-regulated at s2 compared to s1 (Table S1). In contrast, in *A. thaliana* the close homologs *EPFL4* and *EPFL6* have been shown to have distinct functions in inflorescence growth^62,63^. However, two very recent studies revealed that *EPFL4* and *EPFL6* are also expressed in the periphery of the shoot apical meristem (SAM) and function redundantly with *EPFL1* and *EPFL2* to regulate the meristem size and leaf initiation^64,65^. In particular, signaling downstream of EFPL ligands and their receptors confines the WUS-CLV3 pathway to the center of the SAM by inhibiting its expression in the meristem periphery^65^. In this regard, it is especially interesting that *AqEPFL4* came out as one of the most connected hub genes in a module that is strongly associated with s1, suggesting that our results provide further evidence for the role of the EPFLs in the meristem homeostasis.

Perhaps the most surprising observation regarding the green-s1 module network is that one gene, *AqAGO5a* (*Aqcoe5G237200*, homologous to *ARGONAUTE5*, *AtAGO5*, in *A. thaliana*; Fig. S9), was not initially identified as a hub gene since neither its MM nor TS values were above the 90^th^ percentile of distributions (Table S2), but it did come up as a potential hub gene in the network visualization due to its strong connections with all the other hub genes. *AGO* genes encode RNA slicers that are involved in post-transcriptional gene silencing by selectively recruiting microRNAs and siRNAs. In *A. thaliana*, *AtAGO5* physically interacts with *miRNA156* and regulates the phrase transition from the vegetative to reproductive state of the meristem^66^. Other *AtAGO* genes, such as *AtAGO1* and *AtAGO10*, have also been shown to function in establishing polarity in floral organs and regulating meristematic activities in the FM by interacting with *miRNA165/166*^67–71^. Moreover, based on our gene phylogeny (Fig. S9a), the *Aquilegia* genome contains a close paralog of *AqAGO5a*, *Aqcoe2G159700* (*AqAGO5b*), which shares 81.4% of similarity with *AqAGO5a* (Fig. S9b). The expression level of *AqAGO5a* was the highest at s1 and then gradually decreases from s2 to s4, while its paralog *AqAGO5b*, showed very stable expression from s1 to s4 (Fig. S9c). It will thus be intriguing to determine what are the targets of these two close paralogs and the potential functional divergence between them.

### The brown and magenta modules captured different aspects of s4 floral development

Interestingly, among the 56 potential hub genes for the brown-s4 module, only three genes code for transcription factors (TFs), and there seemed to be an over-representation of genes coding for key components of the ubiquitination pathways (Table S2). Six out of the 22 hub genes that exhibit a negative association with s4 encode key components in the SCF E3 ligase complexes, including F-box family proteins (*Aqcoe2G310400*, *Aqcoe7G317700*, *Aqcoe7G317600*), RING/U-box family proteins (*Aqcoe7G340300*, *Aqcoe3G311800*), and HCP-like superfamily protein (*Aqcoe5G357900*). In addition, two hub genes that have positive associations with s4 code for a ubiquitin-related protein (*Aqcoe3G298400*) and a subunit of the COP9 complex (*Aqcoe7G310200*), respectively.

The gene network of the brown-s4 module revealed that most of the identified hub genes are highly interconnected to each other and thus are mostly placed at the periphery of the network (Fig. 7). One hub gene, *Aqcoe7G340300*, however, appeared to have strong connections not only to most of the other hub genes but also to a large number of additional module members. *Aqcoe7G340300* codes for a RING/U-box type protein and its homologs, *DA2* and *GRAIN WIDTH AND WEIGHT2* (*GW2*) have been functionally studied in *A. thaliana*^72^ and rice^73^, respectively. Both DA2 and GW2 have E3 ubiquitin ligase activity and exhibit conserved functions in regulating organ size by restricting cell divisions in both systems^74^. The expression level of *Aqcoe7G340300* steadily declined from s1 to s4 (Fig. S4), and if its function in suppressing cell division in lateral organs is conserved, the low expression level at s4 may indicate the onset of cell proliferation in the floral organs.

In contrast to what was observed for the brown-s4 module, in which most of the hub genes were directly connected to each other, the 14 hub genes for the magenta-s4 module were connected to each other via a number of common non-hub genes in the network (Fig. 8).

Among the magenta-s4 hub genes that have negative MMs, *Aqcoe2G063200* encodes a small ubiquitin-like modifier (SUMO) and is homologous to *SUM2* in *A. thaliana*. Sumoylation mediated by SUMOs is an important post-translational protein modification mechanism in plants and is involved in regulating diverse plant physiological and developmental processes, including flowering time^75^. Interestingly, another hub gene with negative MM, *Aqcoe2G315500*, codes for a copper/zinc superoxide dismutase (CSD) and the protein stability of its *A. thaliana* homolog CSD1 has been shown to be maintained via sumoylation^76^. This relationship was captured in our network analysis, making their function in early floral development an interesting target for further study. In addition, *Aqcoe5G226900*, the *Aquilegia* homolog of *MONOPTEROS* (*AqMP*) is another magenta-s4 hub gene with expression level that was highest at s1 and then steadily declining from s2 to s4. In *A. thaliana*, *MP* is critical to maintaining stem cell homeostasis in the SAM^77^ so this decline is consistent with the cessation of FM proliferation (Fig. 4). On the other hand, the homolog of *Aqcoe2G426800*, *EXCESS MICROSPOROCYTES1* (*EXS*), is expressed during the differentiation of microsporocytes and tapetal cells and controls the somatic and reproductive cell fates in *A. thaliana* anthers^78^. If functional conservation is assumed, the sharp increase in the expression of *Aqcoe2G426800* at s4 (Fig. S4) likely marks the beginning of microsporogenesis in *Aquilegia*.

## Conclusions

The current study is the first in-depth transcriptome profiling of early *Aquilegia* floral development. Similar studies at finely dissected stages of early floral development have only been done in the model species *A. thaliana*^79–81^, although analyses have also been performed in tomato SAMs, and early inflorescences of model monocot species^82,83^. In contrast to the previous *A. thaliana* work, the relatively large size of *Aquilegia* FMs allowed us to visually confirm the developmental stage of dissected meristems and perform a large number of biological replicates. Overall, our results reveal similar themes to the *A. thaliana* analyses, with down-regulation of meristem maintenance corresponding to a shift towards primordium proliferation and differentiation. For the developmental window on which we chose to focus, the morphological changes between successive floral developmental stages are subtle, which helped us to tease apart the dynamic transcriptional changes between the stages. In particular, we have uncovered small numbers of DE genes when comparing s1 to s2 (67), and s2 to s3 (49), with only five genes appearing in common between both DE lists (Table S1). These two groups of DE genes mark two important processes during early floral development: the DE genes between s1/s2 appear to correspond to the termination of the FM and the end of organogenesis, while the DE genes between s2/s3 as associated with the onset of floral organ morphogenesis. Analyzing the homologs of known genes in the FM termination pathway (Fig. 4) provided evidence that the *A. thaliana* mechanisms are likely not conserved in *Aquilegia.* We have also identified a number of key genes that are likely of importance for different stages of early floral development in *A. coerulea*, including *AqPAN*, *AqSAP*, *AqEFLP4*, and *AqAGO5*, as well as many top DE genes and hub genes for which the homologs have not been studied in *A. thaliana*. Thus, our study is the third study to date to indicate that the EPFLs play a previously overlooked role in the meristem homeostasis (Fig. 6), as supported by its identification as one of the core hub genes for the green-s1 module.

In addition, understanding the mechanisms leading to functional divergence of close paralogs is an important component of understanding gene evolution in general. Utilizing DE analysis and WGCNA, we have uncovered evidence of potential functional divergence between the close paralogs of several key genes expressed during early floral development in *Aquilegia*.

These include the close paralogs of *AqSTM1* and *AqSTM2*, *AqUFO1* and *AqUFO2*, and *AqAGO5a* and *AqAGO5b*, of which only *AqSTM2*, *AqUFO1*, and *AqAGO5a* were recovered as core hub genes of the green-s1 module (Fig. 6; Table, S2). These observations help to direct future investigations on the mechanisms and consequences of the functional divergence between close paralogs; for instance, whether or not *AqAGO5a* and *AqAGO5b* function in different tissues or have the same miRNA targets.

Finally, our results highlight the dynamics and potential role of several post-transcriptional and post-translational regulatory mechanisms during early floral development in *A. coerulea*. In terms of post-transcriptional regulation, in our network analysis for the green-s1 module, *AqAGO5a* appeared as a hub gene due to its strong connections to all the other hub genes in the network, suggesting an important role in small RNA regulation in s1 (Fig. 6). The Argonaute protein family is a critical player in the RNA silencing process as an essential component of the RNA-induced silencing complex^84^. Homologs of the *AGO1*/*5*/*10* clade have been studied in *A. thaliana*, rice, tomato, and tobacco, all of which participate in various important aspects in plant development, including regulation of SAM, FM, germ cell development, and stress responses^84–87^. Nonetheless, small RNA regulation has not yet been explored in *Aquilegia*, making *AqAGO5a* a good starting point for further exploration. It is also clear that post-translational regulation via protein stability plays a major role in the transition from primordium initiation to floral organ morphogenesis in the *Aquilegia* FM. This is supported by the ubiquitination-related hub genes of the brown-s4 module coding for RING/U-box proteins, as well as an abundance of hub genes for both the green-s1 and the brown-s4 modules coding for F-box proteins, which are the components of the SCF E3 ligase complex (Table S2). Some of these genes, such as *UFO* and *SAP*, are long known to participate in floral development in *A. thaliana*^36,55,56,60^, but the connection between other F-box coding genes and floral development still needs further investigation. For instance, only recently *BOP2* was discovered to serve as the substrate adaptor in an SCF E3 ligase complex to regulate LFY post-transcriptionally^88^. Sumoylation is another important post-translational regulation mechanism that stabilizes proteins rather than promoting their turnover, and one of the hub genes for the magenta-s4 module codes for a SUMO (Fig. 8; Table S2). SUMOs have been studied in the context of environmental stress, nitrogen assimilation, and flowering time, but little is known about their role in floral development^75^. GO term enrichment analysis showed that genes in the magenta-s4 module are significantly involved in floral organ development (Fig. 5c; Supp. Data 2), including the SUMO hub gene, making it interesting to explore their connections.

## Supplementary information

Supplementary information.

Supplementary Data 1.

Supplementary Data 2.
