# Supplementary material for: Transcriptome profiling and weighted gene co-expression network analysis of early floral development in Aquilegia coerulea
Source: Sci Rep. 2020 Nov 12;10:19637. doi: 10.1038/s41598-020-76750-7 (PMC7665038; doi:10.1038/s41598-020-76750-7)
Supplement: Supplementary file 1 — Supplementary information. [file 41598_2020_76750_MOESM1_ESM.docx]

Transcriptome profiling and weighted gene co-expression network analysis of early floral development in *Aquilegia coerulea*

Ya Min & Elena M. Kramer

**Supplementary information: 9 figures, 2 tables.**


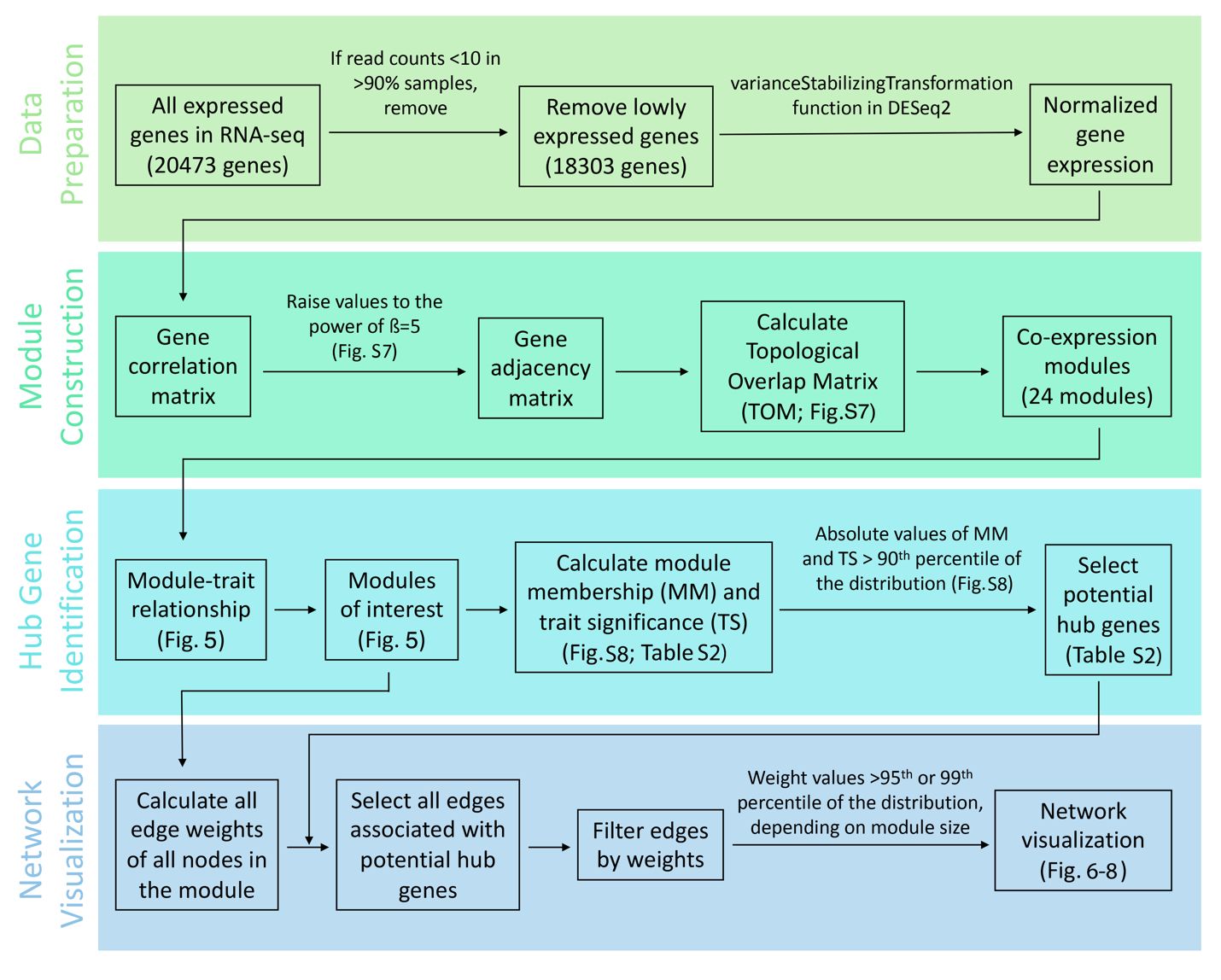


**Figure S1.** Workflow of constructing weighted gene co-expression network and identifying hub genes of modules of interest.


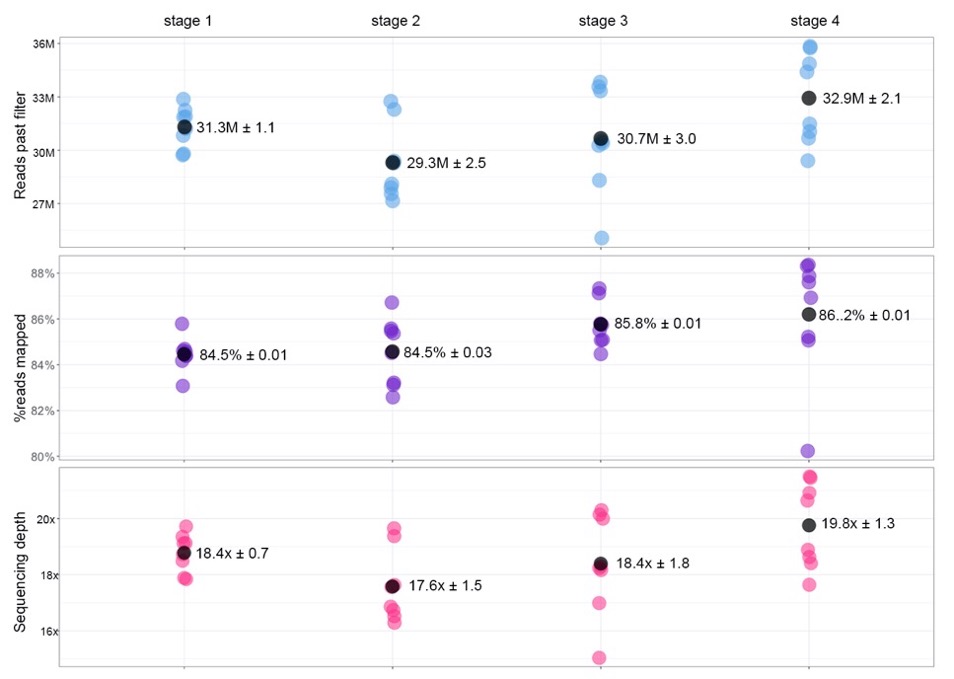


Figure S2. Read statistics and sequencing depths of all 32 libraries in the study. The mean (indicated as a black dot) and the standard deviation of each group were shown as numbers.


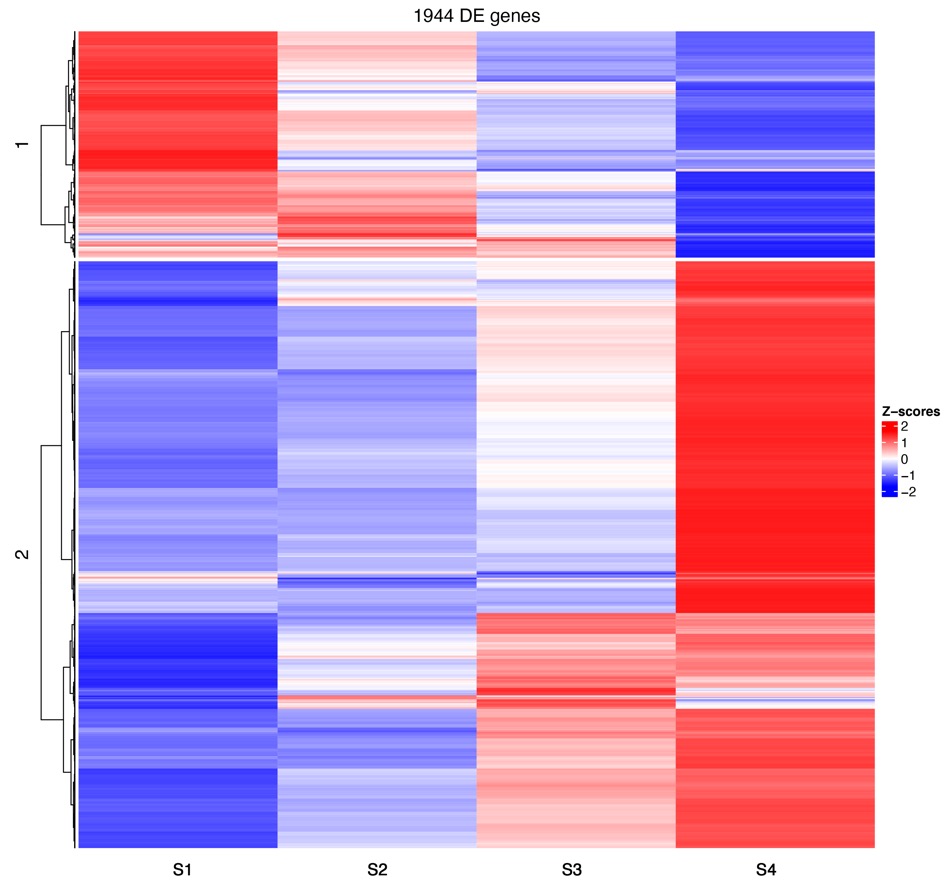


Figure S3. Heatmap of 1944 DE genes. Gene expression across the developmental stages was scaled over developmental as the average expression of all biological replicates per stage subtracted by the average expression of all replicates of all stages, then divided by the standard deviation of the expression of all replicates of all stages.


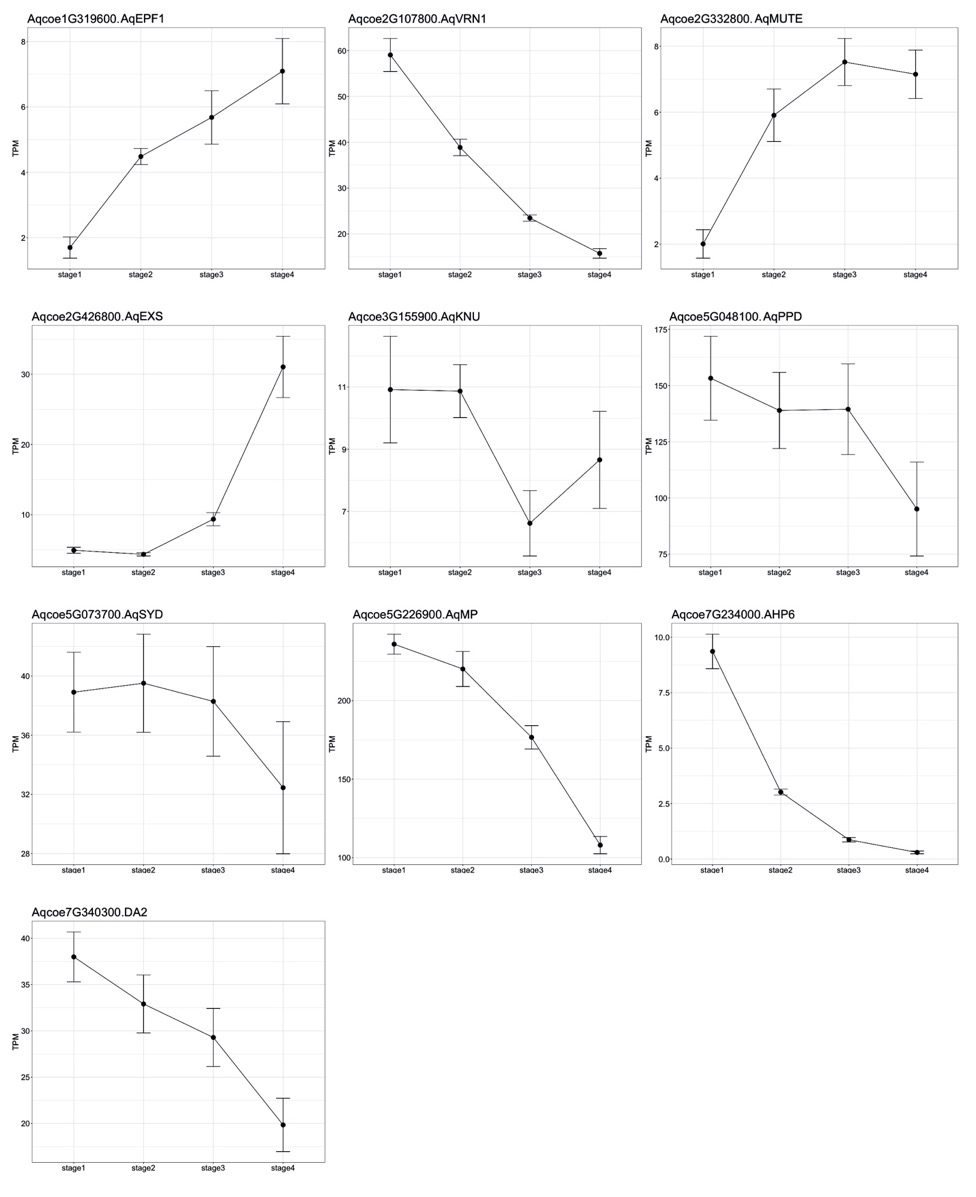


Figure S4. Expression levels of genes of interest from s1 to s4.

**
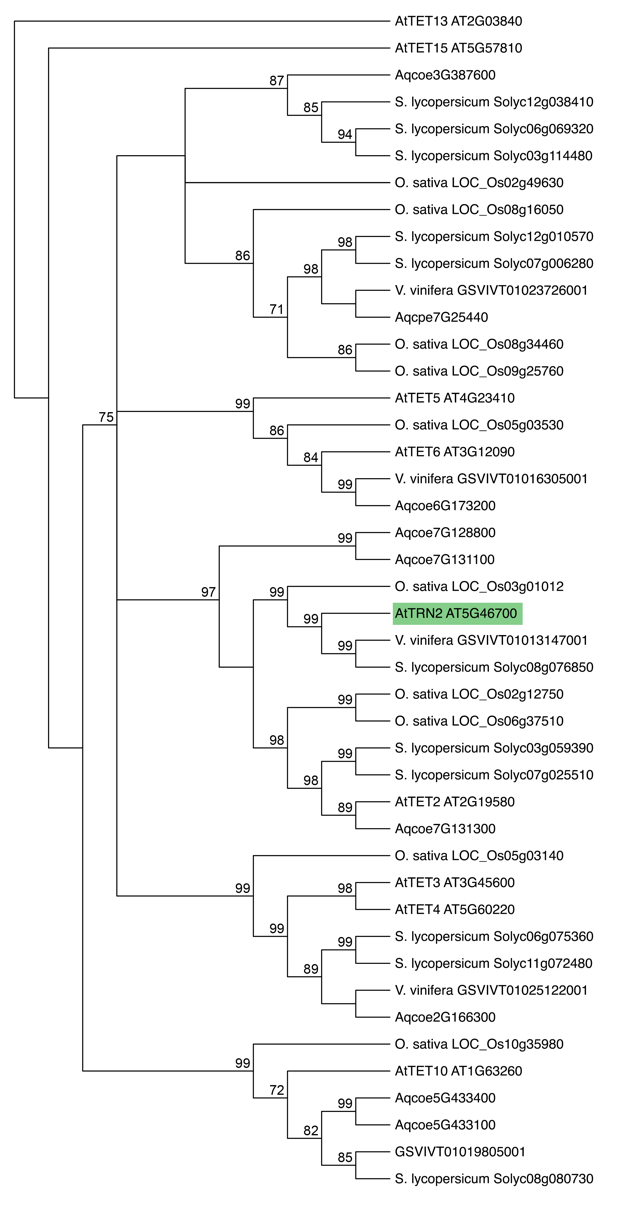
**

Figure S5. Neighbor-joining tree of TRN2. Species included in this phylogeny: *A. coerulea* (all identifiers started with prefix “Aqcoe”), *A. thaliana* (all identifiers started with prefix “At”), *Solanum lycopersicum*, *Oryza sativa*, *Vitis vinifera*. The *A. thaliana TRN2* gene is labeled green.

**
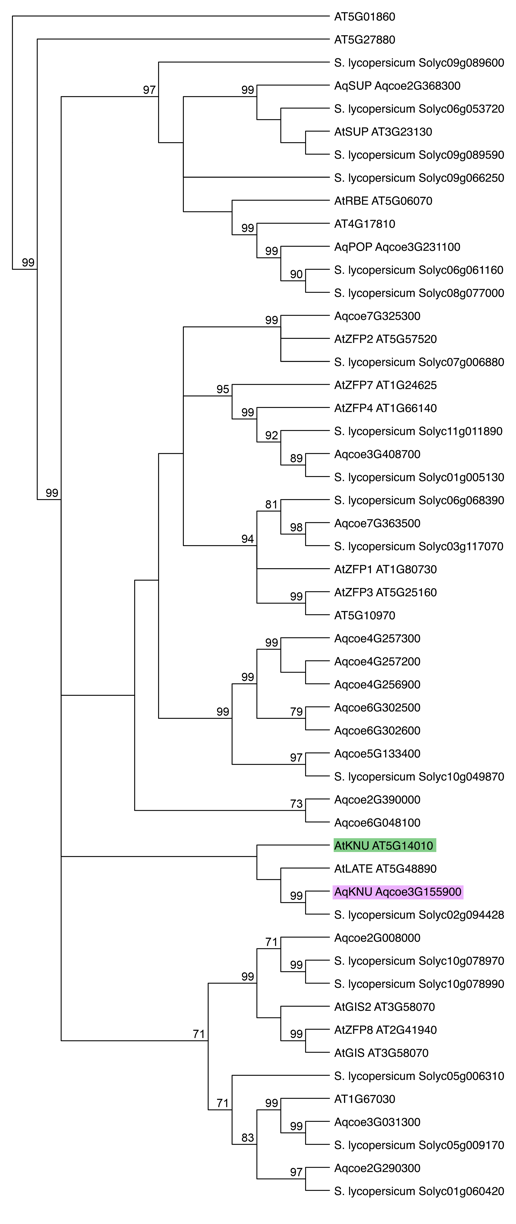
**

Figure S6. Neighbor-joining tree of KNU. Species included in this phylogeny: *A. coerulea* (all identifiers started with prefix “Aqcoe”), *A. thaliana* (all identifiers started with prefix “At”), and *Solanum lycopersicum*. The *A. thaliana* and *A. coerulea* orthologs of *KNU* are labeled green and purple, respectively.


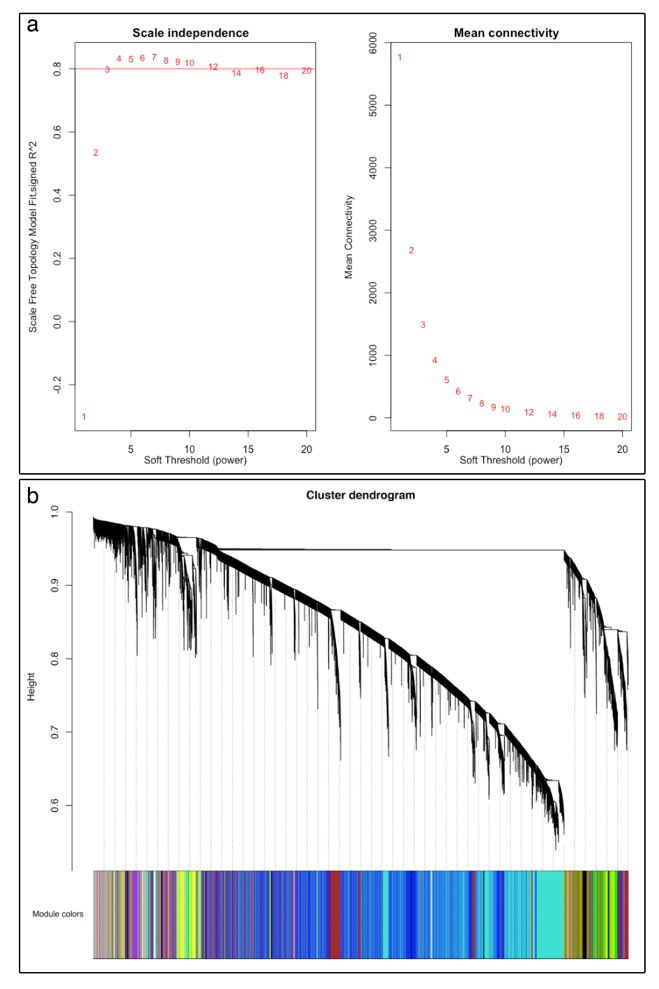


**Figure S7.** Soft threshold and cluster dendrogram used for constructing gene co-expression modules.


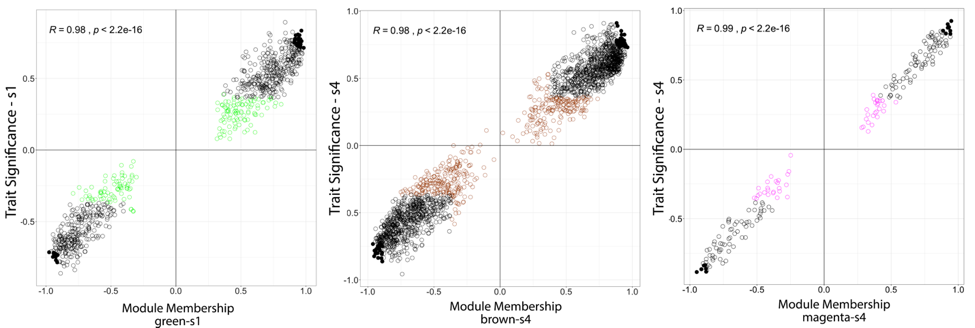
**Figure S8.** Identification of hub genes in modules of interests. Genes in the modules of interest showed strong correlations between their MM (x-axes) and TS (y-axes). In all panels, colored dots are genes that do not have significant p-values associated with MM and/or TS, black circled dots are genes that have significant p-values for both MM and TS, black solid dots are genes that have their MM and TS values above the 90^th^ percentile of the MM and TS of all the genes in the module.


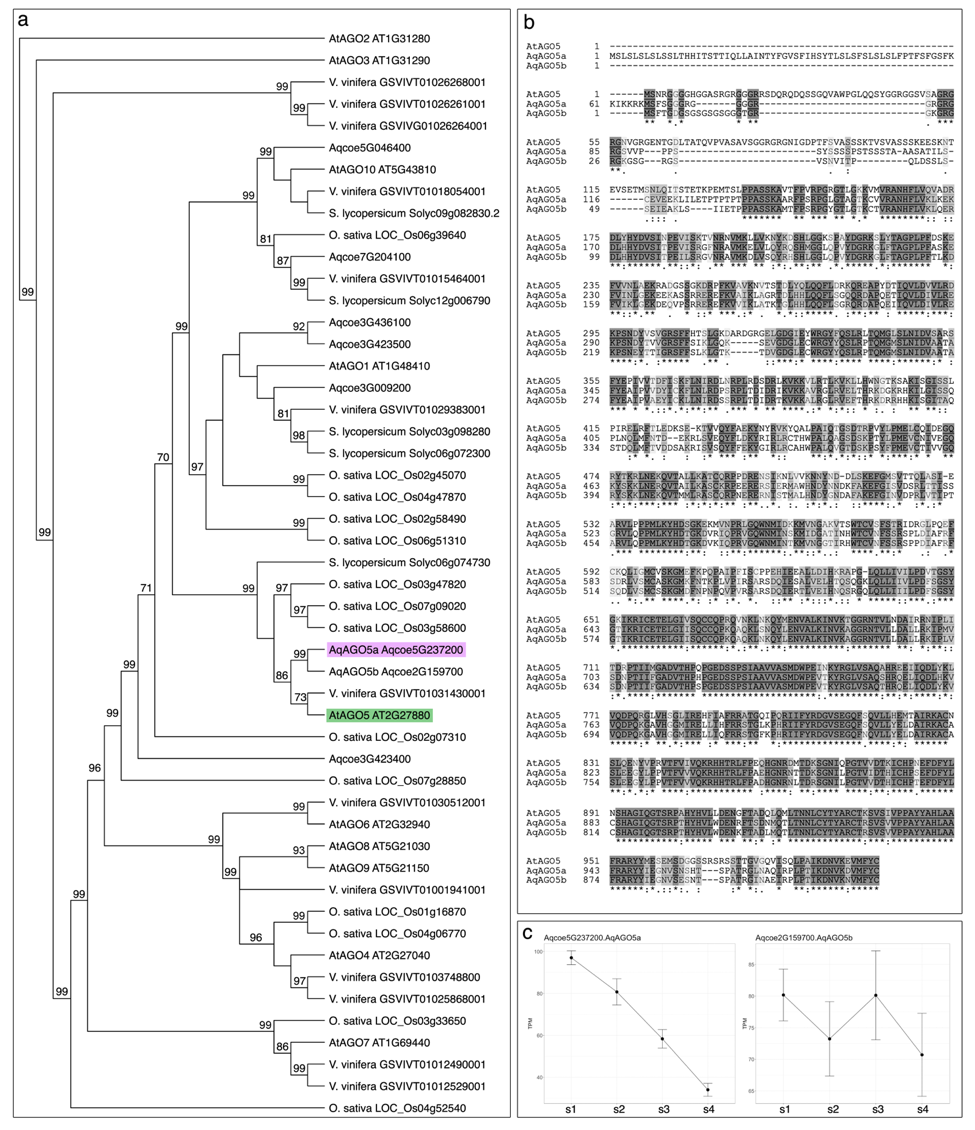


**Figure S9.** Two close paralogs of *AqAGO5* in the *Aquilegia* genome. a. Neighbor-joining tree of *AGO*-like genes. Species included in this phylogeny: *A. coerulea* (all identifiers started with prefix “Aqcoe”), *A. thaliana* (all identifiers started with prefix “At”), *Solanum* *lycopersicum*, *Oryza sativa,* *Vitis vinifera*. The *A. thaliana* and *A. coerulea* genes of interest are labeled green and purple, respectively. b. Alignment of AtAGO5, AqAGO5a, and AqAGO5b showed high sequence similarity (identical and highly conserved sequences are highlighted in the alignment). c. Expression of *AqAGO5a* and *AqAGO5b* over the developmental stages.

Table S1. List of DE genes in s1 vs. s2, and s2 vs. s3. Genes shaded in grey are the genes that appeared DE in both comparisons. Contrast: stages used in the DE comparison; up or down: gene expression change relative to the earlier stages in the comparison; best.hit.tair: Top BLAST hit Arabidopsis gene identifier.

| gene | contrast | adj.pval | up or  down | best.hit.tair | Gene  symbol | annotation |
| --- | --- | --- | --- | --- | --- | --- |
| Aqcoe3G288100 | s1-s2 | 0.000199 | down | AT1G60420.1 |  | DC1 domain-containing protein |
| Aqcoe5G367500 | s1-s2 | 0.000266 | down | AT5G51440.1 |  | HSP20-like chaperones superfamily protein |
| Aqcoe3G106900 | s1-s2 | 0.000435 | down | AT5G14920.1 |  | Gibberellin-regulated family protein |
| Aqcoe4G010700 | s1-s2 | 0.000638 | down | AT3G18080.1 | BGLU44 | B-S glucosidase 44 |
| Aqcoe6G009900 | s1-s2 | 0.001119 | down | AT1G72180.1 |  | Leucine-rich receptor-like protein kinase family |
| Aqcoe3G065300 | s1-s2 | 0.001362 | down | AT1G69180.1 | CRC | Plant-specific transcription factor YABBY family |
| Aqcoe5G158800 | s1-s2 | 0.001379 | down | AT5G53390.1 |  | O-acyltransferase (WSD1-like) family protein |
| Aqcoe7G098000 | s1-s2 | 0.001379 | down | AT1G31770.1 | ABCG14 | ATP-binding cassette 14 |
| Aqcoe1G376900 | s1-s2 | 0.001808 | down | AT3G50660.1 | DWF4 | Cytochrome P450 superfamily protein |
| Aqcoe7G162300 | s1-s2 | 0.003137 | down | AT2G25810.1 | TIP4;1 | tonoplast intrinsic protein 4;1 |
| Aqcoe7G117200 | s1-s2 | 0.00338 | down |  |  |  |
| Aqcoe1G410100 | s1-s2 | 0.003892 | down | AT5G01310.1 | APTX | APRATAXIN-like |
| Aqcoe5G280000 | s1-s2 | 0.004755 | down | AT1G37140.1 | MCT1 | MEI2 C-terminal RRM only like 1 |
| Aqcoe1G456000 | s1-s2 | 0.005392 | down |  |  |  |
| Aqcoe6G058300 | s1-s2 | 0.006734 | down | AT4G39700.1 |  | Heavy metal transport/detoxification superfamily |
| Aqcoe3G383300 | s1-s2 | 0.007649 | down | AT5G38790.1 |  |  |
| Aqcoe2G332800 | s1-s2 | 0.009355 | down | AT3G06120.1 | MUTE | basic helix-loop-helix (bHLH) DNA-binding superfamily |
| Aqcoe5G328500 | s1-s2 | 0.009582 | down | AT1G01630.1 |  | Sec14p-like phosphatidylinositol transfer family |
| Aqcoe7G355700 | s1-s2 | 0.011215 | down | AT5G65730.1 | XTH6 | xyloglucan endotransglucosylase/hydrolase 6 |
| Aqcoe1G251000 | s1-s2 | 0.011497 | down | AT5G44160.1 | NUC | C2H2-like zinc finger protein; highly expressed in roots |
| Aqcoe1G393200 | s1-s2 | 0.016863 | down | AT2G40410.2 |  | Staphylococcal nuclease homologue |
| Aqcoe3G375600 | s1-s2 | 0.017412 | down | AT1G11800.1 |  | endonuclease/exonuclease/phosphatase family |
| Aqcoe1G319600 | s1-s2 | 0.021833 | down | AT2G20875.1 | EPF1 | epidermal patterning factor 1 |
| Aqcoe6G077800 | s1-s2 | 0.022081 | down | AT1G22430.1 |  | GroES-like zinc-binding dehydrogenase family protein |
| Aqcoe1G221000 | s1-s2 | 0.023076 | down | AT5G59070.1 |  | UDP-Glycosyltransferase superfamily protein |
| Aqcoe3G342500 | s1-s2 | 0.02475 | down |  |  |  |
| Aqcoe1G350200 | s1-s2 | 0.026168 | down | AT3G26120.1 | TEL1 | terminal EAR1-like 1 |
| Aqcoe5G278000 | s1-s2 | 0.026229 | down | AT1G77700.1 |  | Pathogenesis-related thaumatin superfamily protein |
| Aqcoe5G195900 | s1-s2 | 0.02652 | down | AT1G49310.1 |  |  |
| Aqcoe5G158700 | s1-s2 | 0.030676 | down | AT5G53390.1 |  | O-acyltransferase (WSD1-like) family protein |
| Aqcoe2G260300 | s1-s2 | 0.031581 | down | AT5G64250.2 |  | Aldolase-type TIM barrel family protein |
| Aqcoe5G391100 | s1-s2 | 0.031581 | down | AT1G13130.1 |  | Cellulase (glycosyl hydrolase family 5) protein |
| Aqcoe6G007700 | s1-s2 | 0.031581 | down | AT1G33811.1 |  | GDSL-like Lipase/Acylhydrolase superfamily protein |
| Aqcoe7G131800 | s1-s2 | 0.031581 | down | AT5G56840.1 |  | myb-like transcription factor family protein |
| Aqcoe5G467300 | s1-s2 | 0.031862 | down | AT4G10850.1 |  | Nodulin MtN3 family protein |
| Aqcoe1G221100 | s1-s2 | 0.032472 | down | AT1G78780.2 |  | pathogenesis-related family protein |
| Aqcoe5G146200 | s1-s2 | 0.037741 | down | AT5G59845.1 |  | Gibberellin-regulated family protein |
| Aqcoe2G035400 | s1-s2 | 0.038708 | down | AT1G02335.1 | GL22 | germin-like protein subfamily 2 member 2 precursor |
| Aqcoe6G070900 | s1-s2 | 0.042213 | down | AT1G09910.1 |  | Rhamnogalacturonate lyase family protein |
| Aqcoe1G180700 | s1-s2 | 0.048632 | down | AT3G14360.1 |  | alpha/beta-Hydrolases superfamily protein |
| Aqcoe1G199600 | s1-s2 | 0.048632 | down | AT4G18170.1 | WRKY28 | WRKY DNA-binding protein 28 |
| Aqcoe5G171900 | s1-s2 | 0.048632 | down | AT5G66940.1 |  | Dof-type zinc finger DNA-binding family protein |
| Aqcoe5G293300 | s1-s2 | 0.048632 | down | AT1G09460.1 |  | Carbohydrate-binding X8 domain superfamily protein |
| Aqcoe7G234000 | s1-s2 | 2.69E-06 | up | AT1G80100.1 | AHP6 | histidine phosphotransfer protein 6 |
| Aqcoe3G399500 | s1-s2 | 3.45E-06 | up | AT1G68640.1 | PAN | bZIP transcription factor family protein |
| Aqcoe6G125400 | s1-s2 | 8.02E-06 | up | AT5G66440.1 |  | tRNA-methyltransferase non-catalytic subunit |
| Aqcoe1G384300 | s1-s2 | 9.22E-06 | up | AT5G35770.1 | SAP | Transducin/WD40 repeat-like superfamily protein |
| Aqcoe5G398700 | s1-s2 | 1.98E-05 | up | AT1G67910.1 |  |  |
| Aqcoe7G438600 | s1-s2 | 1.98E-05 | up | AT1G54050.1 |  | HSP20-like chaperones superfamily protein |
| Aqcoe2G020100 | s1-s2 | 4.28E-05 | up | AT5G13750.1 | ZIFL1 | zinc induced facilitator-like 1 |
| Aqcoe1G196000 | s1-s2 | 0.000134 | up | AT3G18990.1 | VRN1 | AP2/B3-like transcriptional factor family protein |
| Aqcoe6G165500 | s1-s2 | 0.000266 | up | AT1G76420.1 | CUC3 | NAC transcriptional regulator superfamily protein |
| Aqcoe6G092100 | s1-s2 | 0.000279 | up | AT5G10510.2 | AIL6 | AINTEGUMENTA-like 6 |
| Aqcoe5G133100 | s1-s2 | 0.001555 | up |  |  |  |
| Aqcoe4G260600 | s1-s2 | 0.00338 | up | AT1G11260.1 | STP1 | sugar transporter 1 |
| Aqcoe5G190300 | s1-s2 | 0.003544 | up | AT3G50870.1 | HAN | GATA type zinc finger transcription factor family |
| Aqcoe7G365300 | s1-s2 | 0.008501 | up | AT4G22060.1 |  | F-box family protein |
| Aqcoe5G018800 | s1-s2 | 0.011215 | up | AT1G35710.1 |  | Protein kinase family with leucine-rich repeat domain |
| Aqcoe2G305200 | s1-s2 | 0.013856 | up | AT4G13420.1 | AHAK5 | high affinity K+ transporter 5 |
| Aqcoe3G159700 | s1-s2 | 0.015316 | up | AT5G14070.1 | ROXY2 | Thioredoxin superfamily protein |
| Aqcoe5G448600 | s1-s2 | 0.02475 | up | AT5G51970.2 |  | GroES-like zinc-binding alcohol dehydrogenase family |
| Aqcoe5G133000 | s1-s2 | 0.025842 | up | AT2G15020.1 |  |  |
| Aqcoe1G439700 | s1-s2 | 0.02652 | up | AT3G52600.1 | CWINV2 | cell wall invertase 2 |
| Aqcoe5G214900 | s1-s2 | 0.02652 | up | AT2G14960.1 | GH3.1 | Auxin-responsive GH3 family protein |
| Aqcoe5G166400 | s1-s2 | 0.030676 | up | AT4G24480.1 |  | Protein kinase superfamily protein |
| Aqcoe1G101900 | s1-s2 | 0.03389 | up | AT3G55560.1 | AGF2 | AT-hook protein of GA feedback 2 |
| Aqcoe4G188300 | s1-s2 | 0.048498 | up | AT1G61730.1 |  | DNA-binding storekeeper protein-related |
| Aqcoe6G143000 | s2-s3 | 2.89E-08 | down | AT5G25250.1 | FLOT1 | membrane-associated protein family |
| Aqcoe6G158500 | s2-s3 | 0.000154 | down | AT5G49660.1 |  | Leucine-rich repeat transmembrane protein kinase |
| Aqcoe1G115900 | s2-s3 | 0.000293 | down | AT5G03790.1 | HB51 | homeobox 51 |
| Aqcoe2G111100 | s2-s3 | 0.000457 | down | AT3G24500.1 | MBF1C | multiprotein bridging factor 1C |
| Aqcoe3G395700 | s2-s3 | 0.000457 | down | AT1G68810.1 |  | basic helix-loop-helix (bHLH) DNA-binding superfamily |
| Aqcoe3G119000 | s2-s3 | 0.001768 | down | AT5G13700.1 | PAO1 | polyamine oxidase 1 |
| Aqcoe5G438600 | s2-s3 | 0.001768 | down | AT5G51440.1 |  | HSP20-like chaperones superfamily protein |
| Aqcoe7G088000 | s2-s3 | 0.001768 | down |  |  |  |
| Aqcoe7G300700 | s2-s3 | 0.001768 | down | AT2G44480.1 | BGLU17 | beta glucosidase 17 |
| Aqcoe6G055000 | s2-s3 | 0.004383 | down | AT1G01580.1 | FRO2 | ferric reduction oxidase 2 |
| Aqcoe6G104800 | s2-s3 | 0.005623 | down | AT2G16050.1 |  | Cysteine/Histidine-rich C1 domain family protein |
| Aqcoe6G007700 | s2-s3 | 0.005667 | down | AT1G33811.1 |  | GDSL-like Lipase/Acylhydrolase superfamily protein |
| Aqcoe0021s0001 | s2-s3 | 0.006293 | down | AT2G44480.1 | BGLU17 | beta glucosidase 17 |
| Aqcoe4G245900 | s2-s3 | 0.006293 | down | AT1G53420.1 |  | Leucine-rich repeat transmembrane protein kinase |
| Aqcoe5G140900 | s2-s3 | 0.006293 | down | AT3G62290.2 | ARFA1E | ADP-ribosylation factor A1E |
| Aqcoe0412s0001 | s2-s3 | 0.008398 | down |  |  |  |
| Aqcoe4G281800 | s2-s3 | 0.009076 | down | AT5G55370.1 |  | MBOAT (membrane bound O-acyl transferase) family |
| Aqcoe2G397800 | s2-s3 | 0.009095 | down | AT1G60500.1 | DRP4C | Dynamin related protein 4C |
| Aqcoe6G151900 | s2-s3 | 0.009095 | down | AT1G53430.1 |  | Leucine-rich repeat transmembrane protein kinase |
| Aqcoe1G045400 | s2-s3 | 0.011591 | down | AT1G53540.1 |  | HSP20-like chaperones superfamily protein |
| Aqcoe3G231100 | s2-s3 | 0.011591 | down | AT4G17810.1 |  | C2H2 and C2HC zinc fingers superfamily protein |
| Aqcoe3G143300 | s2-s3 | 0.012393 | down | AT1G67910.2 |  |  |
| Aqcoe7G134300 | s2-s3 | 0.013429 | down |  |  |  |
| Aqcoe7G057900 | s2-s3 | 0.013875 | down | AT2G28100.1 | FUC1 | alpha-L-fucosidase 1 |
| Aqcoe5G135200 | s2-s3 | 0.014608 | down | AT5G10770.1 |  | Eukaryotic aspartyl protease family protein |
| Aqcoe1G320100 | s2-s3 | 0.01715 | down | AT1G53540.1 |  | HSP20-like chaperones superfamily protein |
| Aqcoe1G221100 | s2-s3 | 0.017173 | down | AT1G78780.2 |  | pathogenesis-related family protein |
| Aqcoe2G426800 | s2-s3 | 0.018221 | down | AT5G07280.1 | EXS | Leucine-rich repeat transmembrane protein kinase |
| Aqcoe4G079300 | s2-s3 | 0.022866 | down | AT2G33850.1 |  |  |
| Aqcoe6G150000 | s2-s3 | 0.02321 | down | AT1G24020.1 | MLP423 | MLP-like protein 423 |
| Aqcoe5G210700 | s2-s3 | 0.02653 | down | AT2G21140.1 | PRP2 | proline-rich protein 2 |
| Aqcoe7G173900 | s2-s3 | 0.034057 | down | AT2G25810.1 | TIP4;1 | tonoplast intrinsic protein 4;1 |
| Aqcoe3G281400 | s2-s3 | 0.034154 | down | AT1G23390.1 |  | Kelch repeat-containing F-box family protein |
| Aqcoe5G388300 | s2-s3 | 0.034154 | down | AT3G01680.1 |  |  |
| Aqcoe7G016400 | s2-s3 | 0.034731 | down | AT5G41761.1 |  |  |
| Aqcoe1G286700 | s2-s3 | 0.036204 | down | AT1G55790.1 |  | Domain of unknown function (DUF2431) |
| Aqcoe1G041200 | s2-s3 | 0.040968 | down | AT3G47090.1 |  | Leucine-rich repeat protein kinase family protein |
| Aqcoe1G240500 | s2-s3 | 0.045497 | down |  |  |  |
| Aqcoe5G328500 | s2-s3 | 0.045497 | down | AT1G01630.1 |  | Sec14p-like phosphatidylinositol transfer family protein |
| Aqcoe4G014200 | s2-s3 | 0.049082 | down | AT2G30933.1 |  | Carbohydrate-binding X8 domain superfamily protein |
| Aqcoe7G055500 | s2-s3 | 0.000112 | up | AT5G51800.1 |  | Protein kinase superfamily protein |
| Aqcoe3G422300 | s2-s3 | 0.000154 | up | AT1G57820.1 | ORTH2 | Zinc finger (C3HC4-type RING finger) family protein |
| Aqcoe7G234000 | s2-s3 | 0.000161 | up | AT1G80100.1 | HP6 | histidine phosphotransfer protein 6 |
| Aqcoe7G028200 | s2-s3 | 0.000321 | up | AT1G49480.1 | RTV1 | related to vernalization1 1 |
| Aqcoe1G161900 | s2-s3 | 0.000457 | up | AT1G30950.1 | AqUFO1 | F-box family protein |
| Aqcoe7G055600 | s2-s3 | 0.00936 | up | AT1G52540.1 |  | Protein kinase superfamily protein |
| Aqcoe5G398700 | s2-s3 | 0.013518 | up | AT1G67910.1 |  |  |
| Aqcoe3G039100 | s2-s3 | 0.019135 | up | AT3G25905.1 | CLE27 | CLAVATA3/ESR-RELATED 27 |
| Aqcoe6G162300 | s2-s3 | 0.034154 | up | AT3G54700.1 | PHT1;7 | phosphate transporter 1;7 |

Table S2. Hub genes identified for module green, brown and magenta. p.MM and p.TS: the p-values associated with MM and TS of that gene, respectively. TS for genes in module brown and magenta are their associations with s4, and TS for genes in module gene are their association with s1. If a gene codes for a transcription factor (TF), the cell will be labeled “Y” in the “TF?” column; best.hit.tair: Top BLAST hit Arabidopsis gene identifier. AqAGO5a is shaded grey.

| Module | Genes | MM | p.MM | TS | p.TS | TF? | best.hit.tair | Gene symbol |
| --- | --- | --- | --- | --- | --- | --- | --- | --- |
| Brown | Aqcoe7G340300 | -0.9616466 | 1.07E-16 | -0.7332959 | 6.04E-06 |  | AT1G78420.2 | DA2 |
| Brown | Aqcoe2G310400 | -0.9521794 | 1.99E-15 | -0.8242372 | 3.89E-08 |  | AT3G62980.1 | TIR1 |
| Brown | Aqcoe2G129900 | -0.949151 | 4.48E-15 | -0.7821305 | 5.41E-07 | Y | AT3G52910.1 | GRF4 |
| Brown | Aqcoe7G023600 | -0.9463122 | 9.17E-15 | -0.7839813 | 4.88E-07 |  | AT5G41620.1 |  |
| Brown | Aqcoe4G028700 | -0.9425578 | 2.23E-14 | -0.8049137 | 1.41E-07 |  | AT1G53380.1 |  |
| Brown | Aqcoe5G348900 | -0.9387144 | 5.23E-14 | -0.7758043 | 7.64E-07 |  | AT5G50600.1 | HSD1 |
| Brown | Aqcoe5G357900 | -0.9345096 | 1.25E-13 | -0.769978 | 1.04E-06 |  | AT1G67340.1 |  |
| Brown | Aqcoe7G317700 | -0.9254857 | 6.76E-13 | -0.8006882 | 1.83E-07 |  | AT2G17030.1 |  |
| Brown | Aqcoe7G278300 | -0.924967 | 7.40E-13 | -0.7496865 | 2.86E-06 |  |  |  |
| Brown | Aqcoe2G306300 | -0.9222258 | 1.18E-12 | -0.8365215 | 1.58E-08 |  | AT3G21420.1 |  |
| Brown | Aqcoe7G442800 | -0.9213011 | 1.38E-12 | -0.7649158 | 1.35E-06 |  | AT1G53730.1 | SRF6 |
| Brown | Aqcoe5G172800 | -0.9151789 | 3.65E-12 | -0.7569515 | 2.01E-06 | Y | AT5G65590.1 |  |
| Brown | Aqcoe7G204100 | -0.9124635 | 5.50E-12 | -0.7704695 | 1.01E-06 |  | AT5G43810.1 | AGO10 |
| Brown | Aqcoe3G353000 | -0.9114763 | 6.36E-12 | -0.7302617 | 6.90E-06 |  | AT1G18670.1 | IBS1 |
| Brown | Aqcoe4G269800 | -0.909873 | 8.02E-12 | -0.7413823 | 4.20E-06 |  | AT4G27260.1 | GH3.5 |
| Brown | Aqcoe6G072000 | -0.9091747 | 8.87E-12 | -0.7794404 | 6.28E-07 |  | AT5G55150.1 |  |
| Brown | Aqcoe7G317600 | -0.8973065 | 4.33E-11 | -0.8389111 | 1.32E-08 |  | AT2G17030.1 |  |
| Brown | Aqcoe5G016100 | -0.8961225 | 5.02E-11 | -0.7412072 | 4.24E-06 |  | AT1G08600.4 | ATRX |
| Brown | Aqcoe3G311800 | -0.8945204 | 6.11E-11 | -0.807955 | 1.16E-07 |  | AT5G62910.1 |  |
| Brown | Aqcoe7G275200 | -0.8931908 | 7.18E-11 | -0.745065 | 3.55E-06 |  | AT1G05950.1 |  |
| Brown | Aqcoe7G174500 | -0.8928686 | 7.46E-11 | -0.7979391 | 2.17E-07 |  |  |  |
| Brown | Aqcoe7G301900 | 0.89644981 | 4.82E-11 | 0.75723208 | 1.98E-06 |  | AT2G24580.1 |  |
| Brown | Aqcoe3G298400 | 0.8990174 | 3.49E-11 | 0.75019613 | 2.79E-06 |  | AT1G31340.1 | RUB1 |
| Brown | Aqcoe5G351900 | 0.90249581 | 2.22E-11 | 0.73257147 | 6.24E-06 |  | AT5G63510.1 |  |
| Brown | Aqcoe6G280700 | 0.90636317 | 1.32E-11 | 0.73739149 | 5.04E-06 |  | AT5G67500.1 | VDAC2 |
| Brown | Aqcoe5G339900 | 0.90648327 | 1.29E-11 | 0.78217358 | 5.40E-07 |  | AT1G28110.2 | SCPL45 |
| Brown | Aqcoe1G108100 | 0.91030535 | 7.54E-12 | 0.79645328 | 2.37E-07 |  | AT5G02230.2 |  |
| Brown | Aqcoe5G420000 | 0.91163132 | 6.21E-12 | 0.75552647 | 2.16E-06 |  | AT1G62660.1 |  |
| Brown | Aqcoe1G482900 | 0.91238625 | 5.56E-12 | 0.73971053 | 4.54E-06 |  | AT2G37300.2 |  |
| Brown | Aqcoe3G261700 | 0.9134797 | 4.72E-12 | 0.75172898 | 2.59E-06 |  | AT2G44770.1 |  |
| Brown | Aqcoe2G303600 | 0.91632099 | 3.06E-12 | 0.85299802 | 4.18E-09 |  | AT1G07930.1 |  |
| Brown | Aqcoe2G392000 | 0.91781086 | 2.42E-12 | 0.7465597 | 3.31E-06 |  | AT2G19070.1 | SHT |
| Brown | Aqcoe1G354900 | 0.91798731 | 2.36E-12 | 0.78082349 | 5.82E-07 |  | AT4G21105.1 |  |
| Brown | Aqcoe1G025500 | 0.91847147 | 2.18E-12 | 0.75341502 | 2.39E-06 | Y | AT2G41710.1 |  |
| Brown | Aqcoe7G012300 | 0.91877951 | 2.08E-12 | 0.82458695 | 3.80E-08 |  | AT4G23850.1 | LACS4 |
| Brown | Aqcoe7G037100 | 0.920983 | 1.45E-12 | 0.74909795 | 2.94E-06 |  | AT5G23860.1 | TUB8 |
| Brown | Aqcoe1G162600 | 0.92188663 | 1.25E-12 | 0.79545123 | 2.51E-07 |  | AT3G45750.1 |  |
| Brown | Aqcoe5G419900 | 0.9223483 | 1.16E-12 | 0.7435899 | 3.80E-06 |  | AT1G62660.1 |  |
| Brown | Aqcoe2G008200 | 0.92242491 | 1.14E-12 | 0.75531329 | 2.18E-06 |  | AT2G44350.1 | CSY4 |
| Brown | Aqcoe6G208700 | 0.92396011 | 8.81E-13 | 0.76840581 | 1.13E-06 |  | AT2G21170.1 | TIM |
| Brown | Aqcoe1G208600 | 0.92495016 | 7.42E-13 | 0.75555715 | 2.15E-06 |  | AT1G78560.1 |  |
| Brown | Aqcoe3G381900 | 0.92643333 | 5.72E-13 | 0.78202229 | 5.44E-07 |  | AT5G15720.1 | GLIP7 |
| Brown | Aqcoe7G310200 | 0.92776045 | 4.51E-13 | 0.77060368 | 1.01E-06 |  | AT1G71230.1 | AJH2 |
| Brown | Aqcoe1G130700 | 0.93159553 | 2.21E-13 | 0.7536983 | 2.36E-06 |  | AT5G52640.1 | HSP83 |
| Brown | Aqcoe7G346400 | 0.93228157 | 1.94E-13 | 0.7502112 | 2.79E-06 |  | AT1G28280.1 |  |
| Brown | Aqcoe1G476800 | 0.93300618 | 1.68E-13 | 0.76743847 | 1.19E-06 |  | AT5G02600.2 |  |
| Brown | Aqcoe4G003000 | 0.93757489 | 6.66E-14 | 0.7703662 | 1.02E-06 |  | AT3G51280.1 |  |
| Brown | Aqcoe3G070800 | 0.93830888 | 5.70E-14 | 0.78245403 | 5.31E-07 |  | AT5G33370.1 |  |
| Brown | Aqcoe2G178600 | 0.93993774 | 4.01E-14 | 0.82540075 | 3.59E-08 |  | AT3G53620.1 | PPa4 |
| Brown | Aqcoe5G298100 | 0.94125073 | 3.00E-14 | 0.75984394 | 1.74E-06 |  | AT1G10630.1 | ARFA1F |
| Brown | Aqcoe2G373500 | 0.941309 | 2.96E-14 | 0.76329432 | 1.47E-06 |  | AT1G04410.1 |  |
| Brown | Aqcoe1G284000 | 0.94373591 | 1.70E-14 | 0.75676159 | 2.03E-06 |  | AT2G42570.1 | TBL39 |
| Brown | Aqcoe3G268700 | 0.94677483 | 8.18E-15 | 0.73569288 | 5.43E-06 |  | AT3G04710.1 | TPR10 |
| Brown | Aqcoe7G074700 | 0.9503244 | 3.29E-15 | 0.74671361 | 3.29E-06 |  | AT4G22670.1 | HIP1 |
| Brown | Aqcoe6G085000 | 0.95051724 | 3.12E-15 | 0.73765197 | 4.98E-06 |  | AT2G33040.1 | ATP3 |
| Brown | Aqcoe2G379800 | 0.96325602 | 6.06E-17 | 0.73089705 | 6.71E-06 |  | AT2G20420.1 |  |
| Magenta | Aqcoe7G121400 | -0.9487602 | 4.95E-15 | -0.8852387 | 1.80E-10 |  | AT3G07170.1 |  |
| Magenta | Aqcoe1G119200 | -0.9140484 | 4.34E-12 | -0.8649105 | 1.44E-09 |  | AT5G22810.1 |  |
| Magenta | Aqcoe7G052800 | -0.8957816 | 5.23E-11 | -0.8383296 | 1.38E-08 | Y | AT1G62990.1 | AqKXL3 |
| Magenta | Aqcoe2G063200 | -0.8857873 | 1.70E-10 | -0.8356779 | 1.69E-08 |  | AT5G55160.1 | SUMO2 |
| Magenta | Aqcoe2G315500 | -0.8802265 | 3.11E-10 | -0.8618137 | 1.92E-09 |  | AT1G08830.1 | CSD1 |
| Magenta | Aqcoe5G226900 | -0.8784816 | 3.74E-10 | -0.8830259 | 2.30E-10 | Y | AT1G19850.1 | AqMP |
| Magenta | Aqcoe2G397800 | 0.8867262 | 1.53E-10 | 0.85457915 | 3.65E-09 |  | AT1G60500.1 | DRP4C |
| Magenta | Aqcoe6G156100 | 0.90715035 | 1.18E-11 | 0.83424252 | 1.88E-08 |  | AT5G65430.1 | GRF8 |
| Magenta | Aqcoe3G326800 | 0.92111809 | 1.42E-12 | 0.90168685 | 2.47E-11 |  | AT5G62740.1 | HIR1 |
| Magenta | Aqcoe2G426800 | 0.92180792 | 1.27E-12 | 0.87592542 | 4.88E-10 |  | AT5G07280.1 | EXS |
| Magenta | Aqcoe7G421900 | 0.93065557 | 2.64E-13 | 0.88081753 | 2.92E-10 | Y | AT3G22100.1 |  |
| Magenta | Aqcoe1G487700 | 0.94027713 | 3.72E-14 | 0.85378066 | 3.91E-09 | Y | AT2G28550.3 | RAP2.7 |
| Magenta | Aqcoe1G318000 | 0.94072166 | 3.38E-14 | 0.83219133 | 2.19E-08 |  | AT2G14830.1 |  |
| Magenta | Aqcoe5G057900 | 0.94748717 | 6.85E-15 | 0.92500274 | 7.35E-13 |  | AT5G26330.1 |  |
| Green | Aqcoe2G409400 | -0.9755349 | 2.69E-19 | -0.7139132 | 1.37E-05 |  | AT3G25400.1 |  |
| Green | Aqcoe4G281800 | -0.9461437 | 9.55E-15 | -0.7462542 | 3.36E-06 |  | AT5G55370.1 |  |
| Green | Aqcoe3G288200 | -0.9399794 | 3.98E-14 | -0.7166918 | 1.22E-05 |  | AT1G60420.1 |  |
| Green | Aqcoe6G143000 | -0.9396955 | 4.23E-14 | -0.7247044 | 8.76E-06 |  | AT5G25250.1 |  |
| Green | Aqcoe7G051100 | -0.9270612 | 5.11E-13 | -0.7239039 | 9.06E-06 |  | AT1G12570.1 |  |
| Green | Aqcoe5G406000 | -0.9250412 | 7.31E-13 | -0.7461483 | 3.37E-06 |  | AT4G13010.1 |  |
| Green | Aqcoe1G393200 | -0.9229315 | 1.05E-12 | -0.7690893 | 1.09E-06 |  | AT2G40410.2 |  |
| Green | Aqcoe3G331600 | -0.9130456 | 5.04E-12 | -0.7845275 | 4.73E-07 |  | AT3G04710.1 |  |
| Green | Aqcoe7G440900 | -0.9095622 | 8.39E-12 | -0.7334189 | 6.01E-06 |  | AT1G75180.1 |  |
| Green | Aqcoe5G237200 | 0.861425 | 1.98E-09 | 0.631277 | 0.000241 |  | AT2G27880 | AGO5 |
| Green | Aqcoe4G224800 | 0.89800807 | 3.96E-11 | 0.73880422 | 4.73E-06 | Y | AT1G62360.1 | AqSTM1 |
| Green | Aqcoe5G104000 | 0.90974779 | 8.17E-12 | 0.74861704 | 3.00E-06 |  | AT2G23060.1 |  |
| Green | Aqcoe1G258100 | 0.91367004 | 4.59E-12 | 0.76613579 | 1.27E-06 | Y | AT5G60200.1 | TMO6 |
| Green | Aqcoe2G305200 | 0.91684747 | 2.82E-12 | 0.77694696 | 7.19E-07 |  | AT4G13420.1 | HAK5 |
| Green | Aqcoe6G165500 | 0.92150814 | 1.33E-12 | 0.80164223 | 1.73E-07 | Y | AT1G76420.1 | CUC3 |
| Green | Aqcoe1G245800 | 0.92384653 | 8.98E-13 | 0.77454195 | 8.18E-07 | Y | AT2G33880.1 | HB-3 |
| Green | Aqcoe3G065400 | 0.92868252 | 3.81E-13 | 0.74379894 | 3.76E-06 | Y | AT5G15800.1 | AqSEP1 |
| Green | Aqcoe5G340300 | 0.93051165 | 2.71E-13 | 0.72948008 | 7.14E-06 | Y | AT5G50915.1 |  |
| Green | Aqcoe1G178700 | 0.93862085 | 5.34E-14 | 0.73211886 | 6.36E-06 | Y | AT2G34710.1 | ATHB14 |
| Green | Aqcoe1G411100 | 0.93874975 | 5.19E-14 | 0.80624108 | 1.30E-07 | Y | AT1G55580.1 | LAS |
| Green | Aqcoe1G341500 | 0.94382852 | 1.66E-14 | 0.78090759 | 5.79E-07 | Y | AT1G62360.1 | AqSTM2 |
| Green | Aqcoe5G181200 | 0.94551752 | 1.11E-14 | 0.76028153 | 1.71E-06 | Y | AT3G51060.1 | AqSTY2 |
| Green | Aqcoe1G161900 | 0.94640198 | 8.97E-15 | 0.70646352 | 1.84E-05 |  | AT1G30950.1 | AqUFO1 |
| Green | Aqcoe7G354200 | 0.94985903 | 3.72E-15 | 0.74443542 | 3.65E-06 |  | AT5G48930.1 | HCT |
| Green | Aqcoe2G033300 | 0.95067045 | 3.00E-15 | 0.80184251 | 1.71E-07 | Y | AT2G41370.1 | AqBOP |
| Green | Aqcoe1G384300 | 0.95157933 | 2.35E-15 | 0.77187764 | 9.42E-07 | Y | AT5G35770.1 | AqSAP |
| Green | Aqcoe2G352600 | 0.95172148 | 2.26E-15 | 0.7195819 | 1.09E-05 |  | AT4G14723.1 | AqEPFL6 |
| Green | Aqcoe2G107800 | 0.95678744 | 5.21E-16 | 0.7746384 | 8.14E-07 | Y | AT3G18990.1 | AqVRN1 |
| Green | Aqcoe1G457900 | 0.95714219 | 4.67E-16 | 0.73317766 | 6.07E-06 |  | AT4G31380.1 | FLP1 |
| Green | Aqcoe3G280300 | 0.95799583 | 3.58E-16 | 0.71887965 | 1.12E-05 | Y | AT1G23380.1 | AqKXL2 |
| Green | Aqcoe1G205900 | 0.95870235 | 2.86E-16 | 0.73864086 | 4.76E-06 |  | AT4G18130.1 | PHYE |
| Green | Aqcoe3G399500 | 0.96521842 | 2.92E-17 | 0.83327693 | 2.02E-08 | Y | AT1G68640.1 | AqPAN |
| Green | Aqcoe7G028200 | 0.96685774 | 1.54E-17 | 0.76016744 | 1.72E-06 | Y | AT1G49480.1 | RTV1 |
| Green | Aqcoe1G459000 | 0.98240239 | 3.27E-21 | 0.71342883 | 1.40E-05 | Y | AT2G37630.1 | AqAS1 |
